# Alcohol-responsive genes identified in human iPSC-derived neural cultures

**DOI:** 10.1038/s41398-019-0426-5

**Published:** 2019-03-12

**Authors:** Kevin P. Jensen, Richard Lieberman, Henry R. Kranzler, Joel Gelernter, Kaitlin Clinton, Jonathan Covault

**Affiliations:** 1 Department of Psychiatry, Yale University School of Medicine, New Haven, CT 06511 USA; 2 VA Connecticut Healthcare System, West Haven, CT 06516 USA; 3 Alcohol Research Center, Department of Psychiatry, University of Connecticut School of Medicine, Farmington, CT 06030–1410 USA; 4 Center for Studies of Addiction, Department of Psychiatry, Perelman School of Medicine of the University of Pennsylvania, Philadelphia, PA 19104 USA; 5 VISN4 MIRECC, Crescenz VAMC, Philadelphia, PA 19104 USA; 6 Institute for Systems Genomics, University of Connecticut, Storrs, CT 06269 USA

## Abstract

Alcohol use contributes to numerous diseases and injuries. The nervous system is affected by alcohol in diverse ways, though the molecular mechanisms of these effects are not clearly understood. Using human-induced pluripotent stem cells (iPSCs), we developed a neural cell culture model to identify the mechanisms of alcohol’s effects. iPSCs were generated from fibroblasts and differentiated into forebrain neural cells cultures that were treated with 50 mM alcohol or sham conditions (same media lacking alcohol) for 7 days. We analyzed gene expression using total RNA sequencing (RNA-seq) for 34 samples derived from 10 subjects and for 10 samples from 5 subjects in an independent experiment that had intermittent exposure to the same dose of alcohol. We also analyzed genetic effects on gene expression and conducted a weighted correlation network analysis. We found that differentiated neural cell cultures have the capacity to recapitulate gene regulatory effects previously observed in specific primary neural tissues and identified 226 genes that were differentially expressed (FDR < 0.1) after alcohol treatment. The effects on expression included decreases in *INSIG1* and *LDLR*, two genes involved in cholesterol homeostasis. We also identified a module of 58 co-expressed genes that were uniformly decreased following alcohol exposure. The majority of these effects were supported in independent alcohol exposure experiments. Enrichment analysis linked the alcohol responsive genes to cell cycle, notch signaling, and cholesterol biosynthesis pathways, which are disrupted in several neurological disorders. Our findings suggest that there is convergence between these disorders and the effects of alcohol exposure.

## Introduction

Alcohol is commonly consumed worldwide^1^. Although moderate intake of alcohol may have modest health benefits^2,3^, its misuse significantly contributes to numerous diseases and injuries from accidents^1,4^. Alcohol consumption can progress to the development of an alcohol use disorder (AUD). AUD affects nearly 14% of the U.S. population and is characterized by tolerance to alcohol’s effects, continued use despite adverse consequences, and the development of withdrawal symptoms upon reducing alcohol intake^5,6^. Persistent heavy alcohol intake has deleterious effects on the brain including changes in connectivity that are associated with a decline in cognitive abilities^7^, frontal lobe grey matter volume loss^8^, loss of white matter and neurodegeneration^9^, increased risk of early-onset dementia^10^, and decreases in cognitive function even with modest alcohol consumption^11^, among others^9,12–14^. Research suggests that the frontal cortex is particularly vulnerable to the degenerative effects of alcohol with large neurons being primarily affected^8,15^. This population of neurons is also vulnerable in Alzheimer’s disease and normal aging^16,17^. It remains to be fully elucidated how the structural changes in the brain may relate to the development and progression of AUD.

Our understanding of the effects of alcohol at the molecular level in human neural cells is limited, due in part to the challenges associated with obtaining and culturing neural cells from human donors to perform controlled experiments. Differentiation of neural cell cultures from induced pluripotent stem cells (iPSCs)^18^ may provide an in vitro model to examine the effects of alcohol on human neural cells derived from characterized donors, potentially facilitating the identification of novel pathways associated with the effects of alcohol exposure on the brain. Recent work has highlighted the potential of iPSC technologies to study the complex actions of alcohol (for review, see^19,20^). To our knowledge, no report to date has coupled human iPSC neural differentiation with RNA sequencing to explore transcriptome-wide effects of alcohol exposure in vitro.

The goal of the current study was to use RNA-Seq to characterize the effects of alcohol in neural cell cultures derived from iPSCs, which were differentiated into forebrain-type neural cells enriched for glutamatergic neurons. Two cohorts were utilized in the current study; a primary cohort of 34 neural cell cultures (from 10 donor subjects) exposed to alcohol continuously for 7 days, and a secondary cohort consisting of 10 neural cultures (from 5 donor subjects) exposed to a 7-day intermittent exposure to alcohol protocol. Our experimental approach included differential expression analysis and weighted gene co-expression-analysis to identify genes and pathways affected by alcohol exposure. With the bulk of our findings being consistent across two independent experiments, our findings highlight a role for genes involved in cholesterol homeostasis, notch signaling and cell cycle.

## Materials And Methods

### Human-induced pluripotent stem cells (iPSCs)

Fibroblasts obtained from subjects enrolled in studies at the University of Connecticut Health Center (Farmington, CT)^21–23^ were reprogrammed to iPSCs using CytoTune retro- or sendai virus kits (Thermo Fisher Scientific) by the University of Connecticut Stem Cell Core (Farmington, CT) and cultured on irradiated mouse embryonic fibroblasts as we have previously described in detail^24–26^. Fibroblast cultures tested negative for mycoplasma contamination. Informed consent was obtained from all subjects, and the study was approved by the University of Connecticut Health Center Institutional Review Board (project# 06-218S-2 and 08-052-2). Pluripotency of the selected colonies was verified by positive immunocytochemistry staining for SSEA-3/4 and NANOG by the University of Connecticut Stem Cell Core. Donor subjects were diagnosed as alcohol dependent (AD) or control based on DSM-IV criteria. The primary analysis was based on a sample of iPSCs derived from 10 donor subjects (5 control and 5 AD subjects). The primary sample included two clones that were selected from 1 AD subject and one clone that was selected from each of the remaining 9 donor subjects, which yielded 11 independent iPSC lines. The second experiment was based on a set of iPSCs derived from 5 donor subjects (2 control and 3 AD). One of the control donors in the second experiment was also used in the primary experiment. Combined, our sample sets included iPSCs generated from 6 controls and 8 AD donors. The average age of the control donors was 35.3 years and of the AD donors 46.1 years. All donor subjects in the primary and secondary cohorts were male. A validation sample set was used to examine expression of genes identified in the primary sample. This cohort consisted of neural cell lines derived from 7 control and 5 AD donors. RNA from 1 control and 1 AD sample in the validation cohort were also used in the primary RNA sequencing analysis. For the validation sample, the average age of the control donors was 35.3 years and of the AD donors 47.6 years. The validation sample included 2 female donors. A matrix describing the sample donors, sample preparation and analyses is shown in Table S1. Samples originating from the same iPSC clone listed in the table each represent independent neural differentiations (e.g. different dates) from that clone.

### Neural differentiation and Immunocytochemistry

iPSCs were differentiated into neural cell cultures utilizing an embryoid-body-based protocol that we have previously described in detail^24^. In the absence of specific morphogens, the protocol yields forebrain-type neural cell cultures enriched for glutamatergic neurons^27^. Following differentiation and plating onto matrigel-coated glass coverslips, neural cells were cultured and matured for 12 weeks prior to experimentation. Our prior work has demonstrated that 8–12 weeks of growth under this protocol generates neural cultures with functional electrophysiological properties as evidenced by mature action potentials, spontaneous synaptic activity, and expression of ligand-gated ionotropic receptors^24,25^. Neural cell markers were examined in differentiated iPSC lines 12 weeks after plating via immunostaining, as we have described^25^. Cells were fixed in 4% paraformaldehyde, permeabilized using 0.2% Triton X-100 (Sigma-Aldrich), and blocked in 5% donkey serum (Jackson ImmunoResearch). The following primary antibodies were diluted in 5% donkey serum and incubated for 24–48 h at 4 °C: mouse anti-beta III-tubulin (1:500, MAB1637, Millipore), mouse anti-GFAP (1:500, MAB360, Millipore), and rabbit anti-MAP2 (1:500, AB5622, Millipore). Appropriate donkey anti-mouse alexa fluor 594 (1:1000, Life Technologies) and donkey anti-rabbit alexa fluor 488 (1:1000, Life Technologies) secondary antibodies diluted in 3% donkey serum were used prior to mounting in DAPI-containing media for visualization.

### Alcohol treatment

Following 12 weeks of maturation, media was fully replaced with either normal neural differentiation media (henceforth referred to as the sham condition) or media supplemented with 50 mM ethanol. Two experimental protocols were used. In the primary experiment, alcohol-containing or sham neural differentiation media was fully replaced every 24 h (our prior work demonstrated that alcohol concentrations decrease from 50 mM to 18 mM after 24 h of incubation)^24^. In the second experiment, media was fully replaced every 48 h. In both experiments, neural cells were treated with alcohol-containing or sham media for 7 days. The primary cohort consisted of 17 iPSC lines differentiated and exposed to sham or alcohol. This includes iPSC lines derived from 3 control and 3 AD donors that were differentiated into neural cultures on two separate occasions and exposed to sham or alcohol, and 2 control and 3 AD lines differentiated once and exposed to sham or alcohol. The second experiment included of 5 iPSC lines (2 control and 3 AD) differentiated once and exposed to sham or alcohol. Thus, there was one set of experiments (batch 1 and batch 2) that had media (alcohol containing or sham) replaced either every 24 h and a second experiment that had media (alcohol containing or sham) replaced every 48 h. qPCR was used to validate changes in expression of the top three genes identified via RNA sequencing. The validation cohort consisted of 12 iPSC lines derived from 12 donor subjects (7 control and 5 AD) differentiated into neural cultures and exposed to alcohol using procedures described for Batch 1 and Batch 2 (7-day continuous protocol). Material from 1 control and 1 AD samples was used as input for RNA sequencing analysis, while the remaining cell lines were differentiated and treated with alcohol in an independent experiment (Table S1). In prior experiments^24^, we observed an approximately 19-hour half-life for ethanol in these culture conditions (with loss likely due to evaporation), suggesting that ethanol exposure would gradually decrease from 50 to ≈20 mM daily (0.23 to 0.09 mg/dl) for the discovery experimental conditions and for experiment 2, from 50 to ≈8 mM over each 48-hour period.

### RNA sequencing

After alcohol or sham treatment, RNA was extracted using TRIzol Reagent (Thermo Fisher Scientific) per the manufacturer’s protocol. The primary analysis was based on RNA pooled from 136 neural cell cultures, with RNA from 4 wells of a 24-well plate per condition (sham and alcohol-treated) per subject, pooled as input to generate 34 cDNA libraries (17 sham-treated, 17 alcohol-treated) for sequencing. The primary analysis included 24 samples that were ribosomal RNA depleted and 10 samples that were poly(A) enriched (Table S1). We refer to the ribosomal RNA-depleted samples as Batch 1 and the poly(A) enriched samples as Batch 2. The second experiment included RNA from 60 neural cell cultures, with 6 wells of a 24-well plate per condition (sham and alcohol treated), per subject, pooled as input to generate 10 cDNA libraries for sequencing. All of the samples in the second experiment were ribosomal RNA depleted. All RNA samples were treated with DNase I (Thermo Fisher Scientific). RNA Integrity Numbers were assessed prior to library preparation, and they ranged from 6.6 to 9.7 (mean = 8.5) for experiment 1 (Batch1 + Batch2) and ranged from 6.3–10 (mean = 8.97) for experiment 2. Randomly primed cDNA libraries (200- to 500-bp inserts) were prepared and sequenced at the Genomics Core of the Yale Stem Cell Center using Illumina TruSeq chemistry for library preparation and the Illumina HiSeq 2000 platform to generate 100-bp reads. Samples from Batch 1 and from the second experiment were paired end sequenced, while Batch 2 was not. Batch 1 and 2 were each separately aligned to the hg19 version of the human reference genome using TopHat2^28^. The second experiment was aligned to the hg38 genome build using STAR^29^. To increase mapping uniformity among samples in the second experiment, cutadapt was used to remove Illumina adapters from the sequence reads prior to alignment^30^. RNA sequencing data are available via the Sequence Read Archive (SRA accession numbers: SRP154768, SRP154763, SRP154762​).

### Quantitative real-time PCR (qPCR) validation experiment

RNA was extracted from sham and alcohol-treated cultures using TRIzol reagent (Thermo Fisher Scientific) and quantified using a NanoDrop 2000 spectrophotometer (Thermo Fischer Scientific). cDNA was synthesized from 2 μg RNA using a High Capacity cDNA Reverse Transcription kit (Thermo Fisher Scientific) and analyzed by quantitative real-time polymerase chain reaction using an Applied Biosystems 7500 instrument (Thermo Fisher Scientific) and TaqMan Assays-on-Demand (Thermo Fisher Scientific) FAM-labeled probe and primer sets for *INSIG1* (Hs00356479_g1), *LDLR* (Hs01092524_m1), and *F2RL2* (Hs00187982_m1). Expression was quantified relative to a VIC-labeled endogenous control gene *HPRT1* (Hs99999909_m1). cDNA synthesized from RNA extracted from each culture well was assayed in triplicate 20 μL reactions using Gene Expression Master Mix (Thermo Fisher Scientific) per the manufacturer’s protocol. PCR cycles were as follows: 95 °C for 10-min, followed by 40 cycles of 95 °C for 15 sec and 60 °C for 60 sec. A standard curve consisting of a 4-level serial dilution of 100%, 50%, 25%, and 12.5% of sham-treated cDNA from one donor subject was added to each plate and used to determine the relative mRNA expression. Paired- t-tests comparing sham vs. alcohol-treated cultures were used to identify statistical significance (*p* < 0.05). Statistical analysis was conducted on expression values generated relative to the standard curve, and data were then normalized to the sham treatment condition for graphical visualization.

### Variant calling and eQTL analysis

Genetic variants were called from the aligned RNASeq data using a Genome Analysis Tool Kit (GATK) workflow that included ‘SplitNCigarReads’ to improve mapping near exon–intron junctions and base recalibration^31^. Variants were called using GATK HaplotypeCaller for 1000 Genomes Phase 3 variants with hg19 reference genome sequence^31,32^. The variant calling was restricted to reads that mapped uniquely to the genome. The called variants were filtered to exclude multi-allelic variants, variants with read depth <30 and fisher strand values <30.0, and clusters of 3 or more SNPs within a window of 35 bases. PLINK was used to convert VCF files to binary format with a filter to exclude variants with genotype quality (gq) score <30^33^. For each sample pair (sham and alcohol), we used the genetic data from the sample with the highest call rate for eQTL analysis. For eQTL analysis, we focused on Batch 1 samples (ribo-depleted) because Batch 2 (poly(A) enriched) had a small effective sample size (2 unique samples). Variants with minor allele frequency <0.2 and missing in >50 % of the sample were excluded from the eQTL analysis. A principal component analysis examining the SNP variation among the samples showed tight clustering of samples from the same subject relative to other samples (Figure S1). The association of SNPs to gene expression was tested using Matrix eQTL^34^. We focused on identifying “cis” acting eQTLs that were <1000 base pairs from the start and end of a gene based on the gene boundaries defined by the UCSC Genes track of the UCSC genome browser (https://genome.ucsc.edu). Genes that had greater than 2 counts per million reads in at least 13 Batch 1 samples ( >50% of total) were retained for eQTL analyses. Two CPM was roughly 12 counts for the sample with the smallest library size (6.0 M) and 48 counts for the average (24.0 M) library size. Included in the analysis model were treatment condition (sham or alcohol) and a variable representing the paired cultures (1 through 12). SNP effects on gene expression were tested with an additive linear model. A separate model was run to test for SNP by alcohol treatment interactive effects on gene expression. We tested 10,616 SNP–gene pairs for associations. For this and subsequent analyses, sham-treated and alcohol-treated sample pairs were considered independent observations in the statistical models. That is, sample pairs generated from separate iPSC clones from the same subject were considered independent given our prior work showing allelic expression discordance in neural cultures derived from independent iPSC clones from the same donor^26^. This is consistent with the observation that many of the allelic effects on gene expression that occur in somatic cells are randomly reassigned in iPSCs after reprogramming and subsequent neural differentiation^35^.

### Differential expression analysis

To characterize effects on gene expression, we analyzed the number of read counts per gene. For the primary analysis, read counts were determined using HTSeq with uniquely mapped reads and UCSC gene boundary definitions^36^. We used *voom* to test for differential gene expression based on alcohol versus sham treatment and AD case versus control donor status^37^. Genes that had greater than 2 counts per million (CPM) reads in at least 18 samples (>50% of total) were retained for the analysis. Two CPM was roughly 12 counts for the sample with the smallest library size (6.0 million) and 48 counts for the average (24.0 million) library size resulting in a set of 13,258 transcripts. We hypothesized moderate effects on gene expression (fold change > 1.5), which is consistent with our prior work on alcohol’s effects on gene expression in neural cultures derived from iPSC, and with 17 samples per treatment group we had >80% power to detect effects at an FDR threshold of 10%^38,39^. For all analyses, counts were quantile normalized. For the alcohol treatment analysis, the duplicateCorrelation function of *voom* was used to account for correlations between paired cultures (sham and alcohol treated), while batch (ribo depleted (Batch1) or poly(A) enriched (Batch 2)) and treatment (sham or alcohol) were included as fixed effects. Case versus control effects were analyzed using the duplicateCorrelation function to account for correlations between repeated subjects with batch (ribo depleted (Batch1) or poly(A) enriched (Batch 2)), treatment (sham or alcohol), and case or control status included as fixed effects. A similar approach was used to analyze effects in the second experiment. For this analysis, gene counts reported by STAR were analyzed with an inclusion threshold of >3 CPM in >5 samples, which was approximately 40 reads for the average library size (13.0 million), and a total of 12,233 transcripts. The duplicateCorrelation function of *voom* was used to account for correlations between paired samples and treatment (sham vs alcohol) was analyzed as a fixed effect.

### Weighted gene co-expression network analysis methods

We investigated the expression of groups of highly correlated genes using weighted gene co-expression network analysis (WGCNA)^40^. Prior to WCGNA network construction and module detection, Combat was used to adjust for differences between RNA-Seq Batch 1 (ribo-depleted) and Batch 2 (poly(A) enriched)^41^. The following parameters were used for WGCNA: network type = signed, soft power threshold = 14, corFnc = “bicor”, maxPOutliers = 0.05, minModuleSize = 30, and modules were merged at maximum dissimilarity threshold of 0.25. The steps in the module construction are illustrated in Figure S6. Eighteen modules were identified with sizes that ranged from 42 to 3263 genes (Table S5). The module eigengenes were analyzed for association to alcohol treatment using a mixed model that included treatment (sham or alcohol) as a fixed effect and a random effect to account for correlations between paired treatments (sham and alcohol). The threshold for significance was Bonferroni corrected (*p* < 0.003) to account for testing the association of 18 modules for effects of alcohol treatment.

### Pathway and enrichment analyses

Differentially expressed genes (adjusted *p* < 0.1) were evaluated with Ingenuity Pathway Analysis. Canonical Pathways with a Benjamini-Hochberg adjusted p value < 0.1 are reported. For this analysis, the genes were ranked by the log fold change reported by *voom*. DAVID and g:Profiler were used to evaluate the function of genes in the “yellowgreen” module^42,43^. We used the geneSetTest function of the Limma program to test whether gene sets identified in the primary sample were differentially ranked in the secondary sample. We tested for differential ranking of genes associated with alcohol treatment at an adjusted p value < 0.1, for 3 gene sets identified in the primary analysis, which were genes in the Notch Signaling pathway, Superpathway of Cholesterol Biosynthesis pathway and yellowgreen module and for sets of genes reported by a recent study that investigate the effect of alcohol on gene expression in rat brain^44^. We tested for differential ranking of eQTLs identified by GTEx among the eQTLs identified in the iPSC-derived neural cell cultures. For the iPSC-derived neural culture eQTLs results, if a SNP or gene was tested more than once, the SNP or gene with the lowest p value was retained for enrichment testing. This resulted in 4,834 non-redundant SNPs and genes. For tests of differential ranking we assumed a “mixed” effect direction, unless noted otherwise, and used the test statistic for ranking. We tested GTEx eQTLs from 10 brain regions, a non-neural tissue (whole blood), and a larger, composite list based on GTEx eQTLs identified in any tissue^45^. GTEx data were acquired from UCSC Genome Browser Tables (https://genome.ucsc.edu) based on the eQTLs from 44 Tissues from GTEx midpoint release (V6). We limited testing to SNPs that had the same allelic effect direction between iPSC and GTEx datasets. The overlap with GTEx summary data for each eQTL category is shown in Table S7.

## Results

### iPSCs differentiate into frontal cortical-like neural cultures

**Fig. 1:**
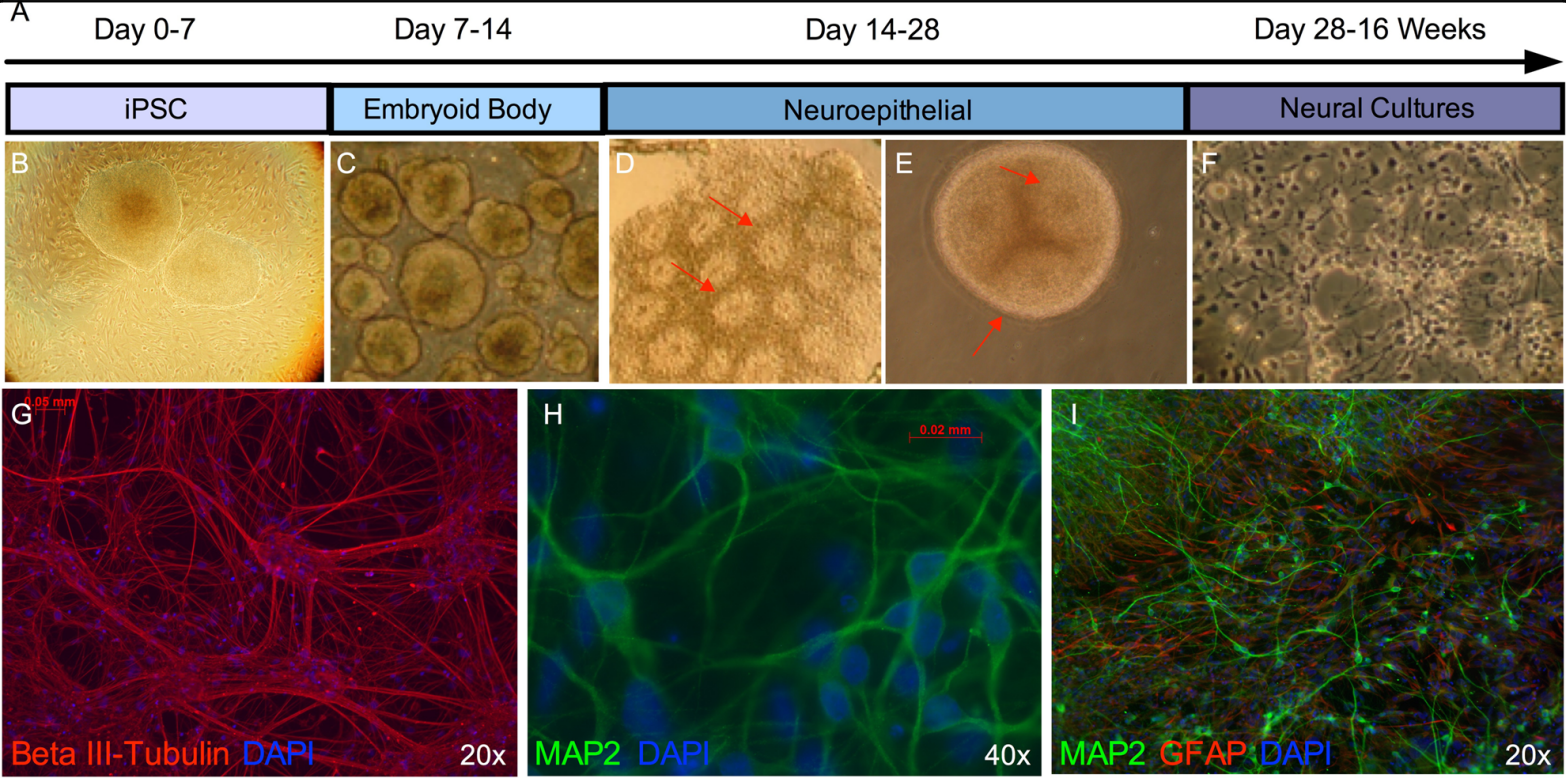
Neural differentiation of human iPSCs. **a** Schematic depicting the neural differentiation protocol. Induced pluripotent stem cells (**b**) are cultured on irradiated mouse embryonic fibroblasts for 7 days, following which they are cultured in suspension for 7 days to allow for the formation of embryoid bodies (**c**). Embryoid bodies are plated on a laminin substrate for 7 days to generate neuroepithelial cells (**d**), which form neural rosette-like structures (indicated by red arrows). Neuroepithelial cells are cultured in suspension for an additional 7 days to form and expand neurospheres (**e**), which display neural rosette-like structures (red arrows), before being manually dissociated and plated onto glass coverslips in neural media (**f**). After 12 weeks in neural media, cultures contain numerous Beta III-tubulin-positive neurites (**g**), pyramidal shaped MAP2-postive neurons (**h**), and GFAP-positive astrocytes (**i**)

We utilized an embryoid-body based differentiation protocol (Fig. 1a–f) to generate mixed neural cultures from human iPSCs generated from control and AD donors. Following 12 weeks of neural maturation, iPSC-derived cultures contained dense Beta III-tubulin positive neurites **(**Fig. 1g), MAP2-positive neurons with pyramidal morphology (Fig. 1h, i), and GFAP-positive astrocytes (Fig. 1i). These findings are consistent with our prior work demonstrating this protocol efficiently generates mixed neural cell cultures with ~50% TBR1-positive glutamate neurons that have the ability to generate trains of action potentials, display spontaneous synaptic activity indicative of synapse formation, and express functional glutamatergic and GABAergic ionotropic receptors, with no difference in the ability of iPSCs from control or AD donors to generate neural cultures^24,25^.

**Fig. 2:**
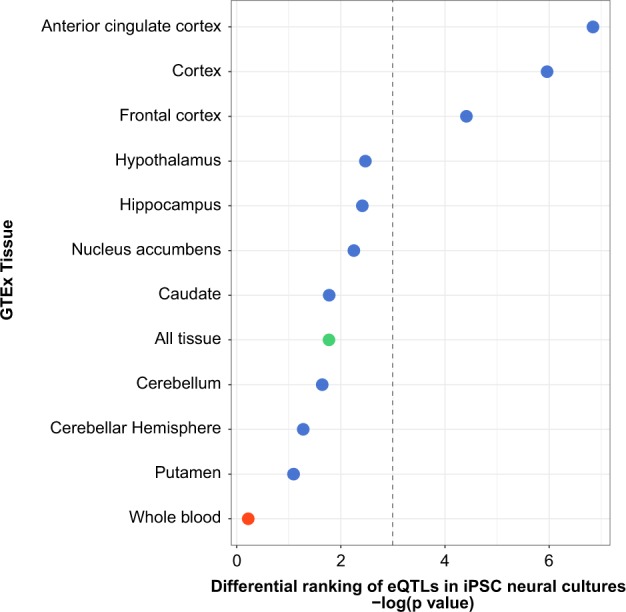
The effects of primary tissue eQTLs in neural cell cultures derived from iPSCs. The differential effects of previously identified eQTLs from 10 brain regions studied by the Genotype-Tissue Expression (GTEx) project. The effects in different brain regions (blue) are contrasted to a non-neuronal tissue, whole blood (red), and a composite set of previously identified eQTLs from 44 different tissues (green). The dashed line marks *p* < 0.05

To validate our neural differentiation, we used SNP information to identify gene regulatory effects in the neural cell cultures and characterized their relationship to effects previously reported for primary neural tissue. There were 14,770 autosomal exonic SNPs identified at a minor allele frequency greater than 20%, and that were genotyped in at least 50% of samples. In total, there were 10,690 SNP–gene association tests. There was 1 SNP-gene expression association significant at an FDR threshold of 5% and 72 SNP-gene associations significant at an FDR threshold of 10%. The top associations are shown in Figure S2. Notably, SNPs that were previously identified as eQTLs in cortex tissue had effects that ranked significantly higher among the eQTLs identified in iPSC-derived neural cultures (anterior cingulate cortex (BA24), *p* = 0.001; cortex, *p* = 0.003; and frontal cortex(BA9)), *p* = 0.012; Fig. 2). In contrast, SNPs that were previously identified as eQTLs from non-neural tissue (whole blood) were not ranked differently (*p* = 0.8). There was also little difference when examining a larger, composite list that included eQTLs previously identified in at least one of 44 tissues. The number of SNPs overlapping between datasets is show in Table S7. These findings indicate that the neural cell cultures have the capacity to recapitulate gene regulatory effects observed in primary tissue with some tissue specificity for frontal cortical regions.

### The effects of alcohol on gene expression in neural cell cultures derived from iPSCs

**Fig. 3:**
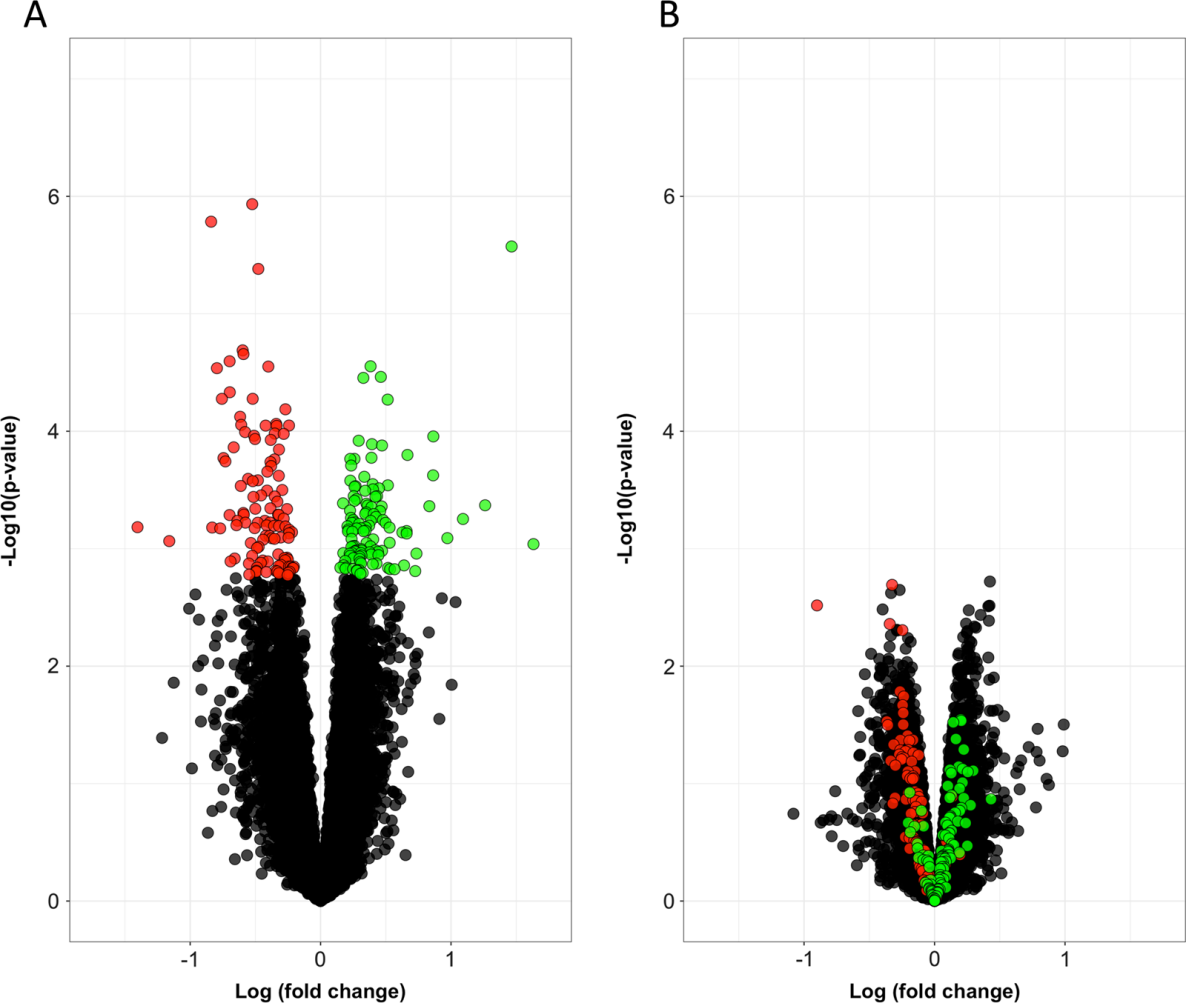
The effects of alcohol treatment on gene expression in neural cell cultures derived from iPSCs. There were 226 genes that were differentially expressed (p adj < 0.1) in the discovery sample. The down (red) and up (green) regulated genes are show in (**a**) the discovery sample (*n* = 17 paired samples) and (**b**) second sample (*n* = 5 paired samples), which had intermittent exposure to the same concentration of alcohol

We tested the effect of alcohol on gene expression following 7 days of alcohol or sham treatment. There were 13,258 transcripts that met our inclusion criteria for testing. Alcohol treatment was associated with a change in the expression of 15 genes (adjusted p value < 0.05) and 226 genes at an adjusted *p* < 0.1. The gene with the top statistically ranked change was *INSIG1*, which encodes the Insulin-induced gene 1 protein. *LDLR*, which encodes the low-density lipoprotein receptor, was the second ranked change. The expression of both genes decreased in response to alcohol (Table 1). We used qPCR to examine the top three alcohol-induced changes in gene expression (*INSIG1*, *LDLR*, and *F2RL2*) in a validation sample set consisting of neural cultures derived from 7 controls and 5 subjects with AD. Among these samples, 1 control and 1 AD donor sample used for validation were included in the RNA sequencing experiment, while the others were from an independent alcohol exposure experiment with conditions that were the same as the Batch 1 and Batch 2 experiments. We observed consistent effects in that alcohol treatment decreased *INSIG1* expression (18% reduction, *t* = 2.79, d*f* = 11, *p* = 0.017), decreased *LDLR* expression (28% reduction, *t* = 3.67, d*f* = 11, *p* = 0.004), and increased *F2RL2* expression (133% increase, *t* = 2.9, d*f* = 11, *p* = 0.015) relative to sham (Figure S4). We also examined the set of 226 differentially expressed genes (adjusted *p* < 0.1) in an independent alcohol treatment RNAseq experiment that included 10 neural cell cultures with intermittent exposure to the same concentration of alcohol. In the second experiment, we observed consistent effects for genes that were either up or down regulated (*p* < 1 × 10^−5^) in the primary sample (Fig. 3). We also used Ingenuity pathway analysis (IPA) to characterize the same set of 226 genes. The top IPA canonical pathway associations are shown in Table S4. The top ranked pathway was Notch Signaling, which included 5 genes. The effect directions for genes in Table S4 are shown in Table 1. There were also several pathway associations related to cholesterol biosynthesis that were anchored on a common set of genes (*DHCR24*, *FDFT1*, *MSMO1*). Neither of the top two differentially expressed genes, *INSIG1* and *LDLR*, contributed to the associations to cholesterol biosynthesis pathways, despite what one might expect given their essential roles in cholesterol homeostasis^46^. As in the discovery sample, genes within the Notch Signaling pathway were uniformly decreased in the second experiment (*p* < 1 × 10^−4^), though genes in the cholesterol pathways and the Molybdenum Cofactor Biosynthesis pathway were not. There were no significant differences in gene expression between neural cell cultures derived from AD and control subjects. There were also no significant SNP by treatment interactive effects (SNP x alcohol) associated with gene expression.

**Table 1:** The effects of alcohol on differentially expressed genes with adjusted *p* < 0.05 and in Ingenuity canonical pathways

Gene	logFC	AveExpr^#^	t	P.Value	adj.P.Val	B
*INSIG1*	−0.52	6.1	−5.83	1.21E-06	0.01	5.32
*LDLR*	−0.84	5.15	−5.74	1.60E-06	0.01	4.95
*F2RL2*	1.47	2.96	5.54	2.97E-06	0.01	3.63
*DHCR24*	−0.48	6.74	−5.43	4.15E-06	0.01	4.23
*DERL3*	−0.59	1.39	−4.91	2.01E-05	0.04	1.72
*C21orf58*	−0.59	3.33	−4.88	2.26E-05	0.04	2.3
*TROAP*	−0.7	3.89	−4.86	2.34E-05	0.04	2.41
*SMAD9*	−0.4	5.26	−4.8	2.85E-05	0.04	2.42
*ALG1*	0.38	4.43	4.79	2.88E-05	0.04	2.31
*AURKB*	−0.8	4.61	−4.78	2.99E-05	0.04	2.32
*ATP6V1C1*	0.33	7.21	4.7	3.82E-05	0.04	2.19
*MDM2*	0.46	7.79	4.7	3.85E-05	0.04	2.17
*FOXM1*	−0.7	4.62	−4.65	4.46E-05	0.04	1.96
*DNMT3B*	−0.52	4.21	−4.6	5.20E-05	0.04	1.78
*TXNRD1*	0.51	8.02	4.6	5.23E-05	0.04	1.89
***DLL1***	−0.46	4.80	−3.95	3.52E-04	0.07	0.17
***NFS1***	0.26	5.67	3.92	3.91E-04	0.07	0.09
***LFNG***	−0.48	3.31	−3.77	6.01E-04	0.07	−0.38
***MOCS2***	0.30	6.49	3.76	6.15E-04	0.07	−0.34
***MFNG***	−0.77	2.17	−3.73	6.72E-04	0.07	−0.63
***MAML1***	−0.22	5.75	−3.70	7.25E-04	0.07	−0.47
***MAML3***	−0.37	4.67	−3.66	8.15E-04	0.08	−0.56
***FDFT1***	−0.35	7.84	−3.66	8.24E-04	0.08	−0.64
***MSMO1***	−0.30	7.00	−3.47	1.37E-03	0.09	−1.08
***FDPS***	−0.23	7.33	−3.46	1.42E-03	0.09	−1.12

#AveExpr in log_2_(counts per million reads)

*Genes in “bold” text are in the pathways shown in Table S4

Genes in the alcohol dehydrogenase gene family involved in the metabolism of ethanol, which are generally expressed at high levels in liver but not brain, where not highly expressed in the neural cell cultures, and 5 (out of 7) were excluded from the primary analysis because of very low expression levels. We conducted a secondary analysis of all mapped genes (no expression threshold), to characterize potential effects from this important gene family. The top effect in the primary and secondary experiment was for *ADH5*. There was a nominal increase in *ADH5* mRNA after alcohol exposure in the primary (unadjusted *p* < 0.05) and secondary (unadjusted *p* < 0.18) samples. *ADH5* was the highest expressed alcohol dehydrogenase gene in each sample, and it was included in the primary analysis. All alcohol dehydrogenase gene effects are shown in Table S8.

We also compared our results from the neural cell cultures derived from iPSCs to a recent study that investigated the effect of alcohol on gene expression in the ventral hippocampus and medial prefrontal cortex of adolescent alcohol-preferring rats^44^. Results (Table S10 and Figure S5) showed that genes that were down regulated in ventral hippocampus were differentially expressed in the same direction in the alcohol-treated neural cell cultures derived from iPSCs (*p* = 1.0 × 10^−6^). Genes that were up regulated in ventral hippocampus had a modest effect in the same direction (*p* = 6.8 × 10^−2^), whereas genes differentially expressed (up or down) in the rodent prefrontal cortex were not differentially expressed in iPSC. A group of 10 genes that had consistent evidence of up regulation in at least 4 out of 11 different rodent brain regions following alcohol exposure were also up regulated following alcohol treatment in the neural cell cultures (*p* = 2.2 × 10^−2^). Among these, the largest effect was for *ATF3* (log FC = 0.43, *p* = 0.012) followed by *BTG2* (log FC = 0.21, *p* = 0.025). *DGKB*, the only gene that was down regulated in at least 4 out of 11 different rodent brain regions following alcohol exposure was unchanged in the neural cell cultures treated with alcohol.

### An alcohol responsive gene co-expression network

**Fig. 4:**
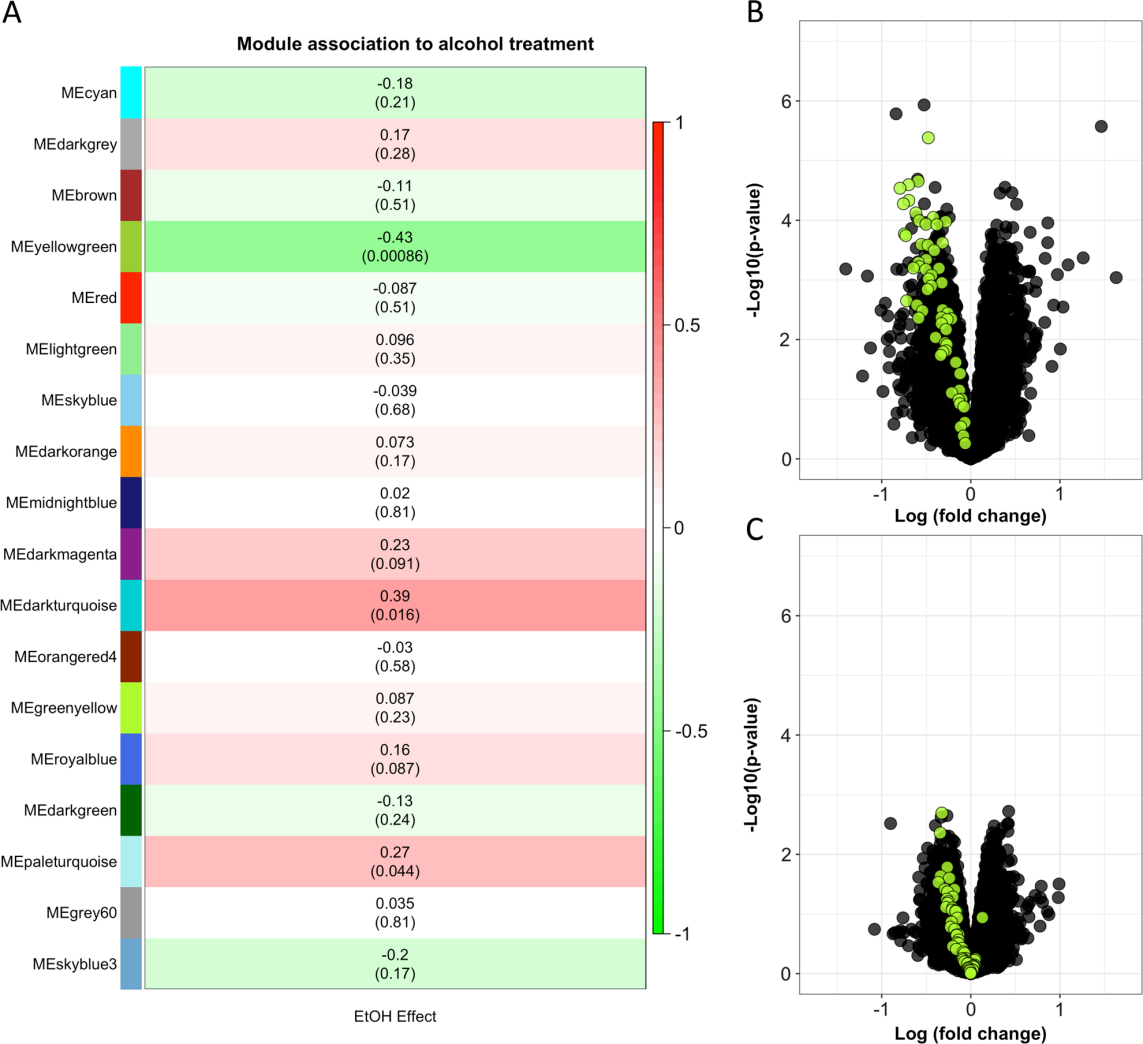
A module of co-expressed genes is down regulated by alcohol treatment. **a** The expression of a module with 58 genes (“yellowgreen”) is lower in the alcohol treatment condition compared to sham. Shown are the Pearson correlations and *p* values for the analysis of module expression to treatment condition. The color is weighted by the magnitude and direction of the Pearson correlation. The response to alcohol for genes in the yellowgreen module is shown in the primary experiment (**b**) and second experiment (**c**)

Using WGCNA, we identified eighteen modules that contained 42 to 3263 co-expressed genes. The module sizes are shown Table S5. The expression of one module, “yellowgreen,” was negatively correlated with the response to alcohol (Pearson r = −0.43, *p* = 0.0009), indicating that the expression of genes within this module was lower in the alcohol condition than the sham condition (Fig. 4b). The 58 genes in the yellowgreen module were nearly uniformly decreased (*p* < 1 × 10^−6^) in the second experiment (Fig. 4c). Within the yellowgreen module, the response to alcohol was correlated with the strength of the association to the module, i.e., genes that were more tightly associated with the module decreased more in the alcohol condition than genes that were less tightly associated to the module (Pearson r = −0.48, *p* = 0.00014) (Figure S7). Genes in the yellowgreen module are shown in Table S6. DAVID analysis indicated that the yellowgreen module was strongly enriched with genes involved in the KEGG Pathway for Cell Cycle (FDR = 1.38 × 10^−4^) and for biological processes such as DNA replication, cell division, DNA repair, and DNA replication initiation (FDR < 3.44 × 10^−4^) (Table S9). Similar annotations were returned with g:Profiler.

## Discussion

Our study used human neural cell cultures derived from iPSCs to characterize the effect of alcohol on gene expression. Our findings, the bulk of which were supported across multiple independent experiments, demonstrate that alcohol affects genes involved in cholesterol homeostasis, notch signaling, and cell cycle pathways. To complement our characterization of the neural cell cultures, we analyzed genetic effects on gene expression to demonstrate that the neural cell cultures have the capacity to recapitulate gene regulatory effects previously identified in primary neural tissues that are relevant to alcohol’s effects. Cholesterol homeostasis, notch signaling and cell cycle pathways are disrupted in several neurological disorders and our findings provide insight into the molecular basis for the potential convergence on these same pathways as a result of alcohol exposure.

Among the top differentially expressed genes following alcohol exposure were *INSIG1* and *LDLR*, which were both down regulated. Prior work has shown that alcohol exposure lowers LDLR levels in mice liver, where LDLR has a critical role in cholesterol turnover^47^. LDLR also has important functions in the brain. For example, LDLR overexpression reduces Aβ aggregation and neuro-inflammatory responses in a mouse model of Alzheimer’s^48^, and LDRL has also been reported to impact learning and memory^49^. Interestingly, *INSIG1* is also involved in regulating cholesterol in the cell, and genetic association studies suggests it has potential links to Alzheimer’s based a reported trend level genomewide association (*p* = 7 × 10^−7^) of an *INSIG1* 3’ untranslated region SNP to Alzheimer’s Disease in a family based association study^46,50^. Along with changes for additional genes involved in cholesterol biosynthesis (Table S4), these findings indicate that alcohol may cause alterations to cholesterol homeostasis within the brain, which might be important clinically given research linking dysregulated cholesterol homeostasis to many neurological disorders, including Alzheimer’s Disease and dementia^51^. Also, cholesterol is a precursor to neuroactive steroids which are thought to mediate some of its behavioral effects via allosteric actions at GABA(A) receptor subtypes^52^. A recent clinical study demonstrated that inhibition of a key enzyme for neuroactive steroid biosynthesis reduces the subjective, sedative effects of acute alcohol intoxication^21^. Reduced sedation in response to alcohol exposure may be a risk factor for the development of AUD^53,54^. Therefore, our finding that alcohol exposure perturbs the expression of genes regulating cholesterol homeostasis may help to explain the relationship between alcohol consumption, the development of AUD, and neurodegeneration.

Our analysis also indicates that several notch signaling pathway genes are affected by alcohol exposure. The notch signaling pathway, which is highly conserved among multicellular organisms, is active in the mammalian adult and developing nervous system. In the adult mammalian nervous system notch pathway genes have an important role in synaptic plasticity^55^, and in a drosophila model, mutations to genes within the notch signaling pathway disrupt the formation of memories for ethanol reward^56^. Given the important role of the notch signaling pathway in determining cell fate during development, our observations might relate to the detrimental effects of alcohol on the adult and/or developing nervous system^57,58^. Likewise, control of the cell cycle and DNA replication, two pathways implicated by our WGCNA approach, are critical in the adult and developing nervous systems. Failure to maintain control of the cell cycle in adult neurons has been linked neurodegenerative disorders^59^. Thus, these gene expression changes could relate to some of clinical observations that heavy alcohol intake is associated with neurodegeneration, a decline in cognitive abilities, and increased risk for early-onset dementia^7–9,12,13^.

Our findings are consistent with some prior studies of alcohol exposure that investigated effects in human and rodent primary neural tissue. For example, genes that were differentially expressed after alcohol exposure in the rat ventral hippocampus had similar effect directions in our human iPSC-derived neural cell cultures treated with alcohol as a set of genes that had consistent effects in multiple different rat brain regions^44^. The McClintick et al. study^37^ also noted that alcohol exposure was associated with the down regulation of several cholesterol pathway genes, although not the same cholesterol pathway genes that we identified here. A study by Lewohl et al. that compared gene expression in post mortem frontal cortex from non-AD and AD subjects showed changes to some genes involved cell cycle regulation, results that are similar to our study’s^60^. These consistent effects are notable given prior studies that demonstrated limited overlap in differentially expressed genes from tissue collected at different developmental stages (e.g., adolescent vs adult) and from tissue from different brain regions at the same developmental stage. For example, in a study by Flatscher-Bader et al.^54^ comparing the nucleus accumbens and ventral tegmental area from AD cases to controls, only 6% of the genes whose expression was associated with AD were shared between the two tissues, and in a rodent study by McBride et al.^55^, there was limited overlap between differentially expressed genes from the nucleus accumbens shell and central nucleus of the amygdala of adolescent rats, and limited overlap when comparing the adolescent effects to effects previously identified in adults for the same tissues^61,62^. The top alcohol dehydrogenase gene effect was *ADH5*. Although *ADH5* encodes a protein with limited alcohol metabolizing activity (K_m_ for ethanol >1000), recent GWAS identified SNPs within *ADH5* associated with alcohol consumption, suggesting that *ADH5* has an important role in mediating the response to alcohol^63,64^. It will be important in future studies to establish and characterize links between what is observed in vitro and in other models of alcohol exposure to the clinical manifestations of AD.

Strengths of our study were the sample size (*n* = 34) and the use of an independent sample (*n* = 10), which was intermittently exposed to the same concentration of alcohol and could therefore reasonably be considered appropriate for replication, and the use of a third experiment to confirm gene expression changes of the top three genes by qPCR. Our experimental design allowed for within-subject comparisons of alcohol effects and our RNA-Seq samples were pools of multiple wells of a culture plate per condition and subject, which likely reduced potential sources of heterogeneity related to individual outliers. However, statistical power was still insufficient to detect certain effects; for example, we did not detect differences between neural cells from AD cases and control subjects, nor did we detect eQTL effects that were modified by alcohol exposure. Additionally, because genetic contributions to alcohol risk appear to be due to the summation of hundreds to thousands of genetic variants of small individual effects, gene expression differences between controls and AD subjects are expected to be much less detectable than the effects of alcohol. Nonetheless, iPSC technology will be useful to characterize neural gene expression correlates for specific AD-associated genotypes. Additionally, the neural culture model may not capture certain epigenetic effects that are important for the response to alcohol (for review, see: Berkel and Pandey (2017))^65^. Indeed, the epigenetic signature of donor somatic cells is largely reset during reprogramming to pluripotency^66^. While methods to retain donor-specific, age-related epigenetic profiles have been successful via direct conversion of fibroblasts into neural cells rather than going through a stem cell state^67^, some epigenetic effects related to the response to alcohol might not be fully recapitulated in cells generated from fibroblasts because they may be more specific to cells in brain tissue. Furthermore, our iPSC differentiation method fails to recapitulate the complex structure and organization of the human brain. Future studies may profitably explore the transcriptomic effects of alcohol exposure on 3D cortical spheroids generated from control and AD donors, which contain multiple functional neural cell types and may better model the effects of alcohol on the human brain^68^. Also, given previous work showing that the gene expression profile of iPSC-derived neural cells more closely resembles that of first trimester human fetal brain^69^, our results suggest that iPSC-derived neural cultures may provide a novel human model system to examine the toxic effects of alcohol exposure early in development, such as in fetal alcohol spectrum disorder. We elected to study the effects of 50 mM ethanol (equivalent blood alcohol concentration = 0.23 mg/dl), a concentration that is commonly observed in individuals with moderate-to-severe AUD, but is higher than that resulting from low-to-moderate alcohol consumption^70,71^. The cells in the second experiment had intermittent exposure to the same dose, and most of the effects on gene expression were consistent between the samples. Thus, the effects of exposure to 50 mM ethanol may to some degree generalize to lower levels of exposure, however additional studies on the effects of lower alcohol doses are warranted.

In conclusion, we used neural cell cultures derived from iPSCs to characterize the effects of alcohol on gene expression. We identified genes and pathways that are affected by exposure to alcohol, including cholesterol homeostasis, notch signaling and cell cycle. These effects point to molecular mechanisms that could contribute to alcohol-induced neurodegeneration. Clinically, alcohol-induced neurodegeneration can be profoundly debilitating, and it can complicate treatment efforts. With support from additional studies, the development of treatments that target these pathways could help to reduce the negative health effects associated with heavy alcohol use.

## Supplementary information

SUPPLEMENTAL TABLES

SUPPLEMENTAL FIGURES

## References

[1] World Health Organization. *Global Status Report On Alcohol And Health 2014*. Report no. 924156475X, WHO Press, Geneva, Switzerland (2014).

[2] O’Keefe JH, Bybee KA, Lavie CJ (2007). Alcohol and cardiovascular health: the razor-sharp double-edged sword. J. Am. Coll. Cardiol..

[3] Gunzerath L, Faden V, Zakhari S, Warren K (2004). National Institute on Alcohol Abuse and Alcoholism report on moderate drinking. Alcohol. Clin. Exp. Res..

[4] Rehm J (2017). The relationship between different dimensions of alcohol use and the burden of disease-an update. Addiction.

[5] Bouchery EE, Harwood HJ, Sacks JJ, Simon CJ, Brewer RD (2011). Economic costs of excessive alcohol consumption in the U.S., 2006. Am. J. Prev. Med..

[6] Grant BF (2015). Epidemiology of DSM-5 alcohol use disorder: results from the national epidemiologic survey on alcohol and related conditions III. JAMA Psychiatry.

[7] Shokri-Kojori E, Tomasi D, Wiers CE, Wang GJ, Volkow ND (2017). Alcohol affects brain functional connectivity and its coupling with behavior: greater effects in male heavy drinkers. Mol. Psychiatry.

[8] Pfefferbaum A, Sullivan EV, Mathalon DH, Lim KO (1997). Frontal lobe volume loss observed with magnetic resonance imaging in older chronic alcoholics. Alcohol. Clin. Exp. Res..

[9] Sutherland GT, Sheedy D, Kril JJ (2014). Neuropathology of alcoholism. Handb. Clin. Neurol..

[10] Schwarzinger M, Pollock BG, Hasan OSM, Dufouil C, Rehm J, QalyDays Study G. (2018). Contribution of alcohol use disorders to the burden of dementia in France 2008-13: a nationwide retrospective cohort study. Lancet Public Health.

[11] Topiwala A (2017). Moderate alcohol consumption as risk factor for adverse brain outcomes and cognitive decline: longitudinal cohort study. BMJ.

[12] Harper C (2009). The neuropathology of alcohol-related brain damage. Alcohol. Alcohol..

[13] Oscar-Berman M, Marinkovic K (2007). Alcohol: effects on neurobehavioral functions and the brain. Neuropsychol. Rev..

[14] Pfefferbaum A (2018). Altered brain developmental trajectories in adolescents after initiating drinking. Am. J. Psychiatry.

[15] Harper C, Kril J (1989). Patterns of neuronal loss in the cerebral cortex in chronic alcoholic patients. J. Neurol. Sci..

[16] Terry RD, Peck A, DeTeresa R, Schechter R, Horoupian DS (1981). Some morphometric aspects of the brain in senile dementia of the Alzheimer type. Ann. Neurol..

[17] Terry RD, DeTeresa R, Hansen LA (1987). Neocortical cell counts in normal human adult aging. Ann. Neurol..

[18] Takahashi K (2007). Induction of pluripotent stem cells from adult human fibroblasts by defined factors. Cell.

[19] Scarnati MS, Halikere A, Pang ZP (2018). Using human stem cells as a model system to understand the neural mechanisms of alcohol use disorders: current status and outlooks. Alcohol.

[20] Prytkova I, Goate A, Hart RP, Slesinger PA (2018). Genetics of Alcohol Use Disorder: A Role for Induced Pluripotent Stem Cells?. Alcohol Clin. Exp. Res.

[21] Covault J (2014). Dutasteride reduces alcohol’s sedative effects in men in a human laboratory setting and reduces drinking in the natural environment. Psychopharmacol. (Berl.).

[22] Milivojevic V, Feinn R, Kranzler HR, Covault J (2014). Variation in AKR1C3, which encodes the neuroactive steroid synthetic enzyme 3alpha-HSD type 2 (17beta-HSD type 5), moderates the subjective effects of alcohol. Psychopharmacology.

[23] Kranzler HR (2014). Topiramate treatment for heavy drinkers: moderation by a GRIK1 polymorphism. Am. J. Psychiatry.

[24] Lieberman R, Levine ES, Kranzler HR, Abreu C, Covault J (2012). Pilot study of iPS-derived neural cells to examine biologic effects of alcohol on human neurons in vitro. Alcohol. Clin. Exp. Res..

[25] Lieberman R, Kranzler HR, Levine ES, Covault J (2017). Examining FKBP5 mRNA expression in human iPSC-derived neural cells. Psychiatry Res..

[26] Lieberman R, Kranzler HR, Joshi P, Shin DG, Covault J (2015). GABRA2 alcohol dependence risk allele is associated with reduced expression of chromosome 4p12 gabaa subunit genes in human neural cultures. Alcohol. Clin. Exp. Res..

[27] Zeng H (2010). Specification of region-specific neurons including forebrain glutamatergic neurons from human induced pluripotent stem cells. PLoS ONE.

[28] Trapnell C, Pachter L, Salzberg SL (2009). TopHat: discovering splice junctions with RNA-Seq. Bioinformatics.

[29] Dobin A (2013). STAR: ultrafast universal RNA-seq aligner. Bioinformatics.

[30] Martin M (2011). Cutadapt removes adapter sequences from high-throughput sequencing reads. EMBnet. journal.

[31] Van der Auwera GA (2013). From FastQ data to high confidence variant calls: the Genome Analysis Toolkit best practices pipeline. Curr. Protoc. Bioinforma..

[32] Genomes Project C (2015). A global reference for human genetic variation. Nature.

[33] Chang CC (2015). Second-generation PLINK: rising to the challenge of larger and richer datasets. Gigascience.

[34] Shabalin AA (2012). Matrix eQTL: ultra fast eQTL analysis via large matrix operations. Bioinformatics.

[35] Jeffries AR (2016). Erasure and reestablishment of random allelic expression imbalance after epigenetic reprogramming. RNA.

[36] Anders S, Pyl PT, Huber W (2015). HTSeq–a Python framework to work with high-throughput sequencing data. Bioinformatics.

[37] Law CW, Chen Y, Shi W, Smyth GK (2014). voom: Precision weights unlock linear model analysis tools for RNA-seq read counts. Genome Biol..

[38] Bi R, Liu P (2016). Sample size calculation while controlling false discovery rate for differential expression analysis with RNA-sequencing experiments. BMC Bioinforma..

[39] Lieberman R, Kranzler HR, Levine ES, Covault J (2017). Examining the effects of alcohol on GABAA receptor mRNA expression and function in neural cultures generated from control and alcohol dependent donor induced pluripotent stem cells. Alcohol.

[40] Langfelder P, Horvath S (2008). WGCNA: an R package for weighted correlation network analysis. BMC Bioinforma..

[41] Leek JT, Johnson WE, Parker HS, Jaffe AE, Storey JD (2012). The sva package for removing batch effects and other unwanted variation in high-throughput experiments. Bioinformatics.

[42] Reimand J (2016). g:Profiler-a web server for functional interpretation of gene lists (2016 update). Nucleic Acids Res..

[43] Huang da W, Sherman BT, Lempicki RA (2009). Systematic and integrative analysis of large gene lists using DAVID bioinformatics resources. Nat. Protoc..

[44] McClintick JN (2018). Gene expression changes in the ventral hippocampus and medial prefrontal cortex of adolescent alcohol-preferring (P) rats following binge-like alcohol drinking. Alcohol.

[45] Consortium GT, Laboratory DA (2017). Coordinating Center -Analysis Working G, Statistical Methods groups-Analysis Working G, Enhancing Gg, Fund NIHC et al. Genetic effects on gene expression across human tissues. Nature.

[46] Yang T (2002). Crucial step in cholesterol homeostasis: sterols promote binding of SCAP to INSIG-1, a membrane protein that facilitates retention of SREBPs in ER. Cell.

[47] Wang Z, Yao T, Song Z (2010). Chronic alcohol consumption disrupted cholesterol homeostasis in rats: down-regulation of low-density lipoprotein receptor and enhancement of cholesterol biosynthesis pathway in the liver. Alcohol. Clin. Exp. Res..

[48] Kim J (2009). Overexpression of low-density lipoprotein receptor in the brain markedly inhibits amyloid deposition and increases extracellular A beta clearance. Neuron.

[49] Mulder M (2004). Low-density lipoprotein receptor-knockout mice display impaired spatial memory associated with a decreased synaptic density in the hippocampus. Neurobiol. Dis..

[50] Hermes ED (2012). Smokeless tobacco use related to military deployment, cigarettes and mental health symptoms in a large, prospective cohort study among US service members. Addiction.

[51] Zhang J, Liu Q (2015). Cholesterol metabolism and homeostasis in the brain. Protein Cell.

[52] Morrow AL, VanDoren MJ, Penland SN, Matthews DB (2001). The role of GABAergic neuroactive steroids in ethanol action, tolerance and dependence. Brain Res. Brain Res. Rev..

[53] King AC, Houle T, de Wit H, Holdstock L, Schuster A (2002). Biphasic alcohol response differs in heavy versus light drinkers. Alcohol. Clin. Exp. Res..

[54] Schuckit MA (1984). Subjective responses to alcohol in sons of alcoholics and control subjects. Arch. Gen. Psychiatry.

[55] Wang Y (2004). Involvement of Notch signaling in hippocampal synaptic plasticity. Proc. Natl Acad. Sci. USA.

[56] Kaun KR, Azanchi R, Maung Z, Hirsh J, Heberlein U (2011). A Drosophila model for alcohol reward. Nat. Neurosci..

[57] Bonthius DJ, West JR (1990). Alcohol-induced neuronal loss in developing rats: increased brain damage with binge exposure. Alcohol. Clin. Exp. Res..

[58] Cartwright MM, Smith SM (1995). Increased cell death and reduced neural crest cell numbers in ethanol-exposed embryos: partial basis for the fetal alcohol syndrome phenotype. Alcohol. Clin. Exp. Res..

[59] Herrup K, Yang Y (2007). Cell cycle regulation in the postmitotic neuron: oxymoron or new biology?. Nat. Rev. Neurosci..

[60] Lewohl JM (2000). Gene expression in human alcoholism: microarray analysis of frontal cortex. Alcohol. Clin. Exp. Res..

[61] Flatscher-Bader T, Harrison E, Matsumoto I, Wilce PA (2010). Genes associated with alcohol abuse and tobacco smoking in the human nucleus accumbens and ventral tegmental area. Alcohol. Clin. Exp. Res..

[62] McBride WJ (2014). Changes in gene expression within the extended amygdala following binge-like alcohol drinking by adolescent alcohol-preferring (P) rats. Pharmacol. Biochem. Behav..

[63] Clarke TK (2017). Genome-wide association study of alcohol consumption and genetic overlap with other health-related traits in UK Biobank (N=112 117). Mol. Psychiatry.

[64] Edenberg HJ (2007). The genetics of alcohol metabolism: role of alcohol dehydrogenase and aldehyde dehydrogenase variants. Alcohol. Res. Health.

[65] Berkel TD, Pandey SC (2017). Emerging role of epigenetic mechanisms in alcohol addiction. Alcohol. Clin. Exp. Res..

[66] Maherali N (2007). Directly reprogrammed fibroblasts show global epigenetic remodeling and widespread tissue contribution. Cell. Stem. Cell..

[67] Mertens J (2015). Directly reprogrammed human neurons retain aging-associated transcriptomic signatures and reveal age-related nucleocytoplasmic defects. Cell. Stem. Cell..

[68] Madhavan M (2018). Induction of myelinating oligodendrocytes in human cortical spheroids. Nat. Methods.

[69] Brennand K (2015). Phenotypic differences in hiPSC NPCs derived from patients with schizophrenia. Mol. Psychiatry.

[70] Pelissier F, Lauque D, Charpentier S, Franchitto N (2014). Blood alcohol concentration in intoxicated patients seen in the emergency department: does it influence discharge decisions?. J. Stud. Alcohol. Drugs.

[71] Olson KN, Smith SW, Kloss JS, Ho JD, Apple FS (2013). Relationship between blood alcohol concentration and observable symptoms of intoxication in patients presenting to an emergency department. Alcohol. Alcohol..

