# Supplementary material for: Alcohol-responsive genes identified in human iPSC-derived neural cultures
Source: Transl Psychiatry. 2019 Mar 12;9:96. doi: 10.1038/s41398-019-0426-5 (PMC6414668; doi:10.1038/s41398-019-0426-5)
Supplement: Supplementary file 1 — SUPPLEMENTAL TABLES [file 41398_2019_426_MOESM1_ESM.pdf]

Table S1: Description of samples and analyses

		Sample Information							Analysis Conducted			
		Subject ID - (Clone #)	*Age*	*Sex*	*Donor Status*	*Preparation*	*Treatments Analyzed*	*Treatment Paradigm*	*Alcohol Effect*	*Case vs. Control*	*WGCNA*	*eQTLs*
Primary Cohort	Batch 1	563-1	38	M	Control	Pol(A) purified	Sham vs. 50mM Alc	7-Day Continuous	X	X	X	X
		563-1	38	M	Control	Pol(A) purified	Sham vs. 50mM Alc	7-Day Continuous	X	X	X	X
		728-7	55	M	AUD	Pol(A) purified	Sham vs. 50mM Alc	7-Day Continuous	X	X	X	X
		704-3	47	M	AUD	Pol(A) purified	Sham vs. 50mM Alc	7-Day Continuous	X	X	X	X
		704-3	47	M	AUD	Pol(A) purified	Sham vs. 50mM Alc	7-Day Continuous	X	X	X	X
	Batch 2	728-4	55	M	AUD	Ribo-depleted	Sham vs. 50mM Alc	7-Day Continuous	X		X	
		728-4	55	M	AUD	Ribo-depleted	Sham vs. 50mM Alc	7-Day Continuous	X		X	
		735-18	45	M	AUD	Ribo-depleted	Sham vs. 50mM Alc	7-Day Continuous	X		X	
		735-18	45	M	AUD	Ribo-depleted	Sham vs. 50mM Alc	7-Day Continuous	X		X	
		716-1	39	M	AUD	Ribo-depleted	Sham vs. 50mM Alc	7-Day Continuous	X		X	
		703-22	44	M	AUD	Ribo-depleted	Sham vs. 50mM Alc	7-Day Continuous	X		X	
		510-2	32	M	Control	Ribo-depleted	Sham vs. 50mM Alc	7-Day Continuous	X		X	
		510-2	32	M	Control	Ribo-depleted	Sham vs. 50mM Alc	7-Day Continuous	X		X	
		542-4	45	M	Control	Ribo-depleted	Sham vs. 50mM Alc	7-Day Continuous	X		X	
		542-4	45	M	Control	Ribo-depleted	Sham vs. 50mM Alc	7-Day Continuous	X		X	
		559-8	32	M	Control	Ribo-depleted	Sham vs. 50mM Alc	7-Day Continuous	X		X	
		534-1	43	M	Control	Ribo-depleted	Sham vs. 50mM Alc	7-Day Continuous	X		X	
Secondary Cohort		520-7	22	M	Control	Ribo-depleted	Sham vs. 50mM Alc	7-Day Intermittent	#		#	
		542-4	45	M	Control	Ribo-depleted	Sham vs. 50mM Alc	7-Day Intermittent	#		#	
		705-1	38	M	AUD	Ribo-depleted	Sham vs. 50mM Alc	7-Day Intermittent	#		#	
		730-8	52	M	AUD	Ribo-depleted	Sham vs. 50mM Alc	7-Day Intermittent	#		#	
		796-1	51	M	AUD	Ribo-depleted	Sham vs. 50mM Alc	7-Day Intermittent	#		#	
qPCR Validation		510-6	32	M	Control	RT-PCR	Sham vs. 50mM Alc	7-Day Continuous	X			
		520-7	22	M	Control	RT-PCR	Sham vs. 50mM Alc	7-Day Continuous	X			
		534-2	43	M	Control	RT-PCR	Sham vs. 50mM Alc	7-Day Continuous	X			
		542-6	45	M	Control	RT-PCR	Sham vs. 50mM Alc	7-Day Continuous	X			
		550-10	35	M	Control	RT-PCR	Sham vs. 50mM Alc	7-Day Continuous	X			
		559-5	32	M	Control	RT-PCR	Sham vs. 50mM Alc	7-Day Continuous	X			
		563-1*	38	M	Control	RT-PCR	Sham vs. 50mM Alc	7-Day Continuous	X			
		704-3*	47	M	AUD	RT-PCR	Sham vs. 50mM Alc	7-Day Continuous	X			
		706-7	47	F	AUD	RT-PCR	Sham vs. 50mM Alc	7-Day Continuous	X			
		716-3	39	M	AUD	RT-PCR	Sham vs. 50mM Alc	7-Day Continuous	X			
		727-8	50	F	AUD	RT-PCR	Sham vs. 50mM Alc	7-Day Continuous	X			
		728-4	55	M	AUD	RT-PCR	Sham vs. 50mM Alc	7-Day Continuous	X			

* Indicates same material used for RNA sequencing and qPCR validation

Table S2: Gene expression changes associated with alcohol treatment.

gene	logFC	AveExpr	t	P.Value	adj.P.Val	B	Entrez ID
INSIG1	-0.5241644	6.07857787	-5.8475869	1.17E-06	0.01089579	5.33047984	3638
LDLR	-0.8391668	5.12620594	-5.7352222	1.64E-06	0.01089579	4.89225427	3949
F2RL2	1.46446651	2.94326731	5.57641905	2.67E-06	0.01180701	3.67380414	2151
DHCR24	-0.4774872	6.71644292	-5.4325765	4.15E-06	0.01374789	4.21753688	1718
DERL3	-0.5973738	1.37328726	-4.9084495	2.05E-05	0.03850426	1.69243715	91319
C21orf58	-0.5913755	3.31303722	-4.8843019	2.20E-05	0.03850426	2.28109751	54058
TROAP	-0.6974945	3.8727077	-4.8380086	2.54E-05	0.03850426	2.30431689	10024
ALG1	0.38317066	4.41135154	4.80524271	2.80E-05	0.03850426	2.30604025	56052
SMAD9	-0.4006334	5.23622693	-4.803565	2.81E-05	0.03850426	2.41097827	4093
AURKB	-0.7947579	4.58525046	-4.7930949	2.90E-05	0.03850426	2.31861232	9212
MDM2	0.46181213	7.77106943	4.73653509	3.45E-05	0.03882208	2.28055736	4193
ATP6V1C1	0.32774115	7.19177469	4.72998748	3.51E-05	0.03882208	2.26333286	528
FOXM1	-0.6961812	4.60112288	-4.6364994	4.66E-05	0.04457534	1.90701837	2305
DNMT3B	-0.5205707	4.1918787	-4.5938078	5.29E-05	0.04457534	1.73919923	1789
KIF18B	-0.756767	3.55416082	-4.5936674	5.30E-05	0.04457534	1.62753793	146909
TXNRD1	0.51433948	8.0047362	4.5884457	5.38E-05	0.04457534	1.86834404	7296
PAPD7	-0.2696596	6.04797842	-4.5250801	6.50E-05	0.05072752	1.69659682	11044
KIFC1	-0.6166582	5.40223317	-4.4761872	7.53E-05	0.05150407	1.54873688	3833
RCOR1	-0.3403072	6.13655729	-4.4292325	8.66E-05	0.05150407	1.43857435	23186
SMOC1	-0.6087237	6.55056477	-4.4230584	8.82E-05	0.05150407	1.42169623	64093
PTMA	-0.2420905	7.27764903	-4.4185505	8.94E-05	0.05150407	1.40718033	5757
POC1A	-0.4212821	3.97700601	-4.4180859	8.95E-05	0.05150407	1.26936367	25886
BRD1	-0.336817	5.43213459	-4.4163568	9.00E-05	0.05150407	1.39085503	23774
CDC20	-0.578833	5.01785536	-4.3754911	0.00010163	0.05150407	1.26673095	991
SCD	-0.3515012	8.80487949	-4.3658421	0.00010459	0.05150407	1.24924783	6319
FADS1	-0.2827253	7.35460809	-4.3635491	0.0001053	0.05150407	1.25292722	3992
UCP2	-0.5102826	4.96328483	-4.3511716	0.00010924	0.05150407	1.20035396	7351
SRXN1	0.86429033	5.46625985	4.34684104	0.00011066	0.05150407	1.2015065	140809
TACC3	-0.5027341	5.60152348	-4.3294708	0.00011651	0.05150407	1.16529266	10460
NRM	-0.3819328	4.13152873	-4.3236842	0.00011853	0.05150407	1.05618549	11270
ZNF436	0.29194158	6.87284216	4.31831433	0.00012043	0.05150407	1.13546088	80818

TMEM199	0.39369454	5.55214279	4.29544292	0.00012887	0.05310957	1.07252358	147007
TIGAR	0.47059665	5.45681153	4.28683728	0.00013219	0.05310957	1.04706295	57103
PKN3	-0.6662786	2.35076215	-4.2745023	0.00013711	0.05346301	0.62643348	29941
ZNF395	-0.3191487	5.98437393	-4.2597804	0.0001432	0.05424528	0.98377803	55893
PTCHD4	0.66603508	3.5393669	4.22390713	0.0001592	0.05614743	0.70043051	442213
CA12	0.38989982	6.82904477	4.20529421	0.00016817	0.05614743	0.83100201	771
MYBL2	-0.7445854	5.15651294	-4.2031205	0.00016925	0.05614743	0.82304137	4605
TM7SF3	0.25952716	6.89825166	4.19766547	0.00017199	0.05614743	0.80943316	51768
EI24	0.22761938	7.36996814	4.19750287	0.00017208	0.05614743	0.80374784	9538
REEP4	-0.3535511	2.73371724	-4.1944394	0.00017363	0.05614743	0.51438958	80346
IQGAP3	-0.729794	5.17754224	-4.1810026	0.00018064	0.05639374	0.76542967	128239
CDC25B	-0.3841471	6.24699292	-4.1767612	0.0001829	0.05639374	0.76146044	994
VPS33A	0.23455318	5.57627732	4.15338853	0.0001959	0.05831309	0.70041172	65082
BCOR	-0.3794815	6.63280994	-4.149894	0.00019792	0.05831309	0.68441769	54880
RNF122	-0.4079475	4.4888605	-4.1123774	0.00022094	0.06367824	0.55756463	79845
NQO1	0.8626441	6.72932585	4.08764655	0.00023752	0.06608733	0.52099721	1728
SNRPA	-0.320381	5.82277629	-4.0848147	0.00023949	0.06608733	0.5218661	6626
KTN1	0.33513986	8.77112989	4.07808419	0.00024425	0.06608733	0.46670613	3895
GTSE1	-0.5555544	4.26005031	-4.0641678	0.00025438	0.06657127	0.41799761	51512
ASF1B	-0.4806092	3.4287707	-4.0544759	0.00026168	0.06657127	0.30281733	55723
PFDN1	0.22828288	6.40053053	4.0521218	0.00026348	0.06657127	0.43096497	5201
CIT	-0.5201534	5.715131	-4.0487015	0.00026612	0.06657127	0.42767571	11113
PINX1	0.3996734	3.89865568	4.02829682	0.00028243	0.06761455	0.28957091	54984
CROT	0.51566817	6.26652527	4.02084157	0.00028863	0.06761455	0.34984822	54677
PLEKHH2	-0.6119669	4.87276531	-4.0163876	0.00029239	0.06761455	0.33299803	130271
PEX3	0.26866963	5.7232767	4.01440499	0.00029408	0.06761455	0.33875742	8504
FBXO22	0.2603427	5.41465113	4.01055946	0.00029739	0.06761455	0.32820176	26263
BCAR3	0.44781007	4.42184979	4.00060002	0.00030613	0.06761455	0.26468973	8412
SPRED3	0.40167526	4.53823903	3.99374134	0.00031229	0.06761455	0.25496282	399473
RPL10	-0.2940418	6.83513662	-3.9891581	0.00031647	0.06761455	0.26006361	6134
RAD54L	-0.4099615	4.61470437	-3.9852604	0.00032008	0.06761455	0.24413896	8438
GPATCH4	0.34124703	5.28858715	3.9839516	0.00032129	0.06761455	0.25837116	54865
DLL1	-0.4563635	4.79806585	-3.9526325	0.00035184	0.06964575	0.17032667	28514

TOMM34	0.25389824	5.57374869	3.94757422	0.00035703	0.06964575	0.16650881	10953
SLC25A20	0.43300358	4.73503454	3.94749738	0.00035711	0.06964575	0.15028422	788
PTMS	-0.3513374	7.67874151	-3.9458155	0.00035885	0.06964575	0.13147816	5763
TMC7	0.42062875	3.12893038	3.94395999	0.00036078	0.06964575	-0.0150252	79905
IGDCC3	-0.5150063	4.19492877	-3.9423539	0.00036246	0.06964575	0.11271294	9543
SCFD2	0.27603302	5.12621099	3.92760968	0.00037826	0.07164187	0.11265132	152579
NFS1	0.26031075	5.66532661	3.91617106	0.00039097	0.07247094	0.08520096	9054
MMAB	-0.3315109	5.00899988	-3.9104516	0.00039748	0.07247094	0.06868605	326625
AP3M2	0.17214577	6.52888194	3.8991501	0.00041066	0.07247094	0.0244181	10947
UTP15	0.35204732	5.12021682	3.89634394	0.00041399	0.07247094	0.03329021	84135
HMOX1	1.26137274	3.92450389	3.88566871	0.00042693	0.07247094	-0.0672175	3162
EDA2R	0.36042765	5.39964336	3.88411143	0.00042885	0.07247094	0.00438795	60401
TRPM8	0.8337179	2.88716958	3.87944371	0.00043466	0.07247094	-0.2070899	79054
GPR19	0.46805487	3.82744783	3.87763135	0.00043693	0.07247094	-0.0840699	2842
MFSD1	0.39046472	6.18636911	3.87359024	0.00044204	0.07247094	-0.0328276	64747
CREB3L4	-0.3846865	3.82328758	-3.8650725	0.00045301	0.07247094	-0.1052776	148327
PLK1	-0.5005975	4.87088711	-3.8618821	0.00045719	0.07247094	-0.0557518	5347
SHMT1	-0.2573951	4.19844976	-3.8598407	0.00045988	0.07247094	-0.0904611	6470
PARN	0.22557613	6.37228903	3.85009127	0.00047295	0.07247094	-0.099301	5073
ABHD4	0.45639221	6.32815578	3.84632455	0.0004781	0.07247094	-0.1065593	63874
SNAPC5	0.34937351	3.75776076	3.83741531	0.00049049	0.07247094	-0.1734633	10302
TK1	-0.5922238	2.8956503	-3.8329231	0.00049685	0.07247094	-0.2781393	7083
DOLK	0.34221427	3.96045087	3.82717673	0.00050511	0.07247094	-0.189673	22845
AEN	0.40764065	4.91189712	3.82561211	0.00050738	0.07247094	-0.1474723	64782
HJURP	-0.5882812	4.84507432	-3.8220792	0.00051255	0.07247094	-0.155944	55355
NXN	-0.3260235	5.73451147	-3.8215589	0.00051331	0.07247094	-0.1590946	64359
GSE1	-0.3213148	7.50391518	-3.8208131	0.00051441	0.07247094	-0.2039185	23199
RCOR2	-0.6978792	6.08252822	-3.8190327	0.00051704	0.07247094	-0.1720128	283248
UBXN8	0.4248137	4.69220935	3.80610998	0.00053654	0.07247094	-0.2022059	7993
KIF3B	0.23362721	7.71927436	3.79959068	0.00054665	0.07247094	-0.262182	9371
RCC2	-0.2826756	6.50840914	-3.794805	0.00055418	0.07247094	-0.2461241	55920
HGD	1.09174817	2.71625158	3.79206177	0.00055855	0.07247094	-0.4337467	3081
LACTB2	0.47856936	3.99057667	3.78481919	0.00057023	0.07247094	-0.2906846	51110

TOR1A	0.25039898	5.83530454	3.78450949	0.00057074	0.07247094	-0.2546433	1861
FBLN1	-0.6375476	6.1739553	-3.7798404	0.0005784	0.07247094	-0.2750678	2192
PRR11	-0.4274194	5.47284865	-3.7792913	0.00057931	0.07247094	-0.2628757	55771
GBA	0.38451125	4.68278841	3.77919147	0.00057948	0.07247094	-0.2677241	2629
ZMAT3	0.26119262	7.09377693	3.76888285	0.00059679	0.07247094	-0.3293952	64393
EFS	-0.3883707	4.8336697	-3.7679549	0.00059837	0.07247094	-0.2902313	10278
PIF1	-0.5760571	2.69896303	-3.7669194	0.00060014	0.07247094	-0.4580162	80119
LFNG	-0.4814411	3.31234782	-3.7665085	0.00060084	0.07247094	-0.3848229	3955
CHAC2	0.49839575	2.28936566	3.76594103	0.00060182	0.07247094	-0.5428126	494143
OGFOD1	0.23955156	6.45059631	3.76565479	0.00060231	0.07247094	-0.3197915	55239
MOCS2	0.29598742	6.49252765	3.7584702	0.00061478	0.07247094	-0.3383693	4338
ESPL1	-0.645041	4.38966616	-3.7494329	0.00063082	0.07247094	-0.3473768	9700
EMP2	-0.413306	5.29888802	-3.7479468	0.0006335	0.07247094	-0.3397844	2013
RRM2B	0.37210208	6.32442359	3.74647765	0.00063615	0.07247094	-0.36469	50484
KIF22	-0.3567272	6.07013897	-3.7448786	0.00063906	0.07247094	-0.3628244	3835
FBL	-0.3185906	7.30908664	-3.743115	0.00064227	0.07247094	-0.3945116	2091
ZCCHC17	0.20595481	6.80774263	3.7419422	0.00064442	0.07247094	-0.38939	51538
TGIF2	-0.2689435	4.80525863	-3.7403447	0.00064736	0.07247094	-0.3596496	60436
OTOF	-1.4036771	0.73872828	-3.7357372	0.00065591	0.07247094	-0.8531711	9381
AP4S1	0.34589235	4.73955238	3.73492003	0.00065743	0.07247094	-0.3756919	11154
MYO15A	-0.8312088	1.54724629	-3.7333678	0.00066034	0.07247094	-0.7290425	51168
MCF2	0.52871034	4.13875896	3.73333115	0.00066041	0.07247094	-0.4063174	4168
CDCA8	-0.5053999	4.8599991	-3.7291181	0.00066837	0.07247094	-0.3865026	55143
DUSP14	0.30979248	4.1116219	3.72875159	0.00066907	0.07247094	-0.4140844	11072
MFNG	-0.7695829	2.1734903	-3.7274591	0.00067153	0.07247094	-0.629771	4242
GATB	0.27642969	5.72733199	3.72703609	0.00067234	0.07247094	-0.3983221	5188
RNF185	0.21264725	6.02669378	3.7192065	0.00068747	0.07297013	-0.42588	91445
DAZAP1	-0.2404209	5.89481957	-3.7171949	0.00069141	0.07297013	-0.427959	26528
HSPH1	0.34493515	7.75335809	3.71108807	0.00070351	0.07297013	-0.4925278	10808
RNLS	0.65938102	2.03000179	3.70886323	0.00070797	0.07297013	-0.7105184	55328
OSTM1	0.33004831	5.78203939	3.70780029	0.00071011	0.07297013	-0.4479703	28962
STAMBP	0.20943029	6.63884898	3.70716169	0.0007114	0.07297013	-0.4759205	10617
DTNA	0.24322064	7.86971747	3.70486964	0.00071604	0.07297013	-0.5101491	1837

MAML1	-0.2191278	5.74645966	-3.7006878	0.00072459	0.07297013	-0.4670614	9794
NOV	0.62708819	4.24116659	3.69975406	0.00072651	0.07297013	-0.4801142	4856
EEF1G	-0.2427864	7.78709717	-3.6929869	0.00074058	0.07342741	-0.5268144	1937
SERPINB8	0.65982931	3.85829881	3.69224762	0.00074214	0.07342741	-0.5237155	5271
CCNF	-0.4203384	4.06426951	-3.6762659	0.00077651	0.07599132	-0.5383331	899
CECR2	-0.3911696	5.78839978	-3.6748991	0.00077952	0.07599132	-0.5340631	27443
DZIP1L	-0.3063174	3.30324114	-3.6641748	0.00080352	0.07708795	-0.6244555	199221
RPS11	-0.2456115	8.41998148	-3.6626753	0.00080694	0.07708795	-0.6086568	6205
UPP1	0.97196801	4.20482174	3.65990673	0.00081328	0.07708795	-0.5791371	7378
MAML3	-0.3736063	4.66949446	-3.659175	0.00081496	0.07708795	-0.5606401	55534
FDFT1	-0.3529156	7.83589842	-3.6554467	0.00082359	0.07708795	-0.6371263	2222
ANXA7	0.23721376	7.07155248	3.6545623	0.00082565	0.07708795	-0.6215341	310
NDUFAF6	0.40304555	5.08109454	3.65142976	0.00083299	0.07720165	-0.5777948	137682
CDCA5	-0.4406846	4.81496015	-3.6490877	0.00083852	0.07720165	-0.5835995	113130
ACTG2	-1.1602324	3.62868373	-3.6393569	0.00086186	0.0788037	-0.6545361	72
GCLM	0.52933842	5.17229274	3.62797795	0.00088995	0.08014009	-0.6358194	2730
MICAL1	-0.5345106	6.41310406	-3.6261912	0.00089445	0.08014009	-0.6750654	64780
ENDOD1	0.35176295	5.71004306	3.62612635	0.00089461	0.08014009	-0.6517338	23052
METTL7B	1.63191861	0.75646597	3.61831884	0.0009145	0.08137174	-1.0999074	196410
DHX29	0.26069654	6.91340542	3.60589956	0.00094701	0.08319944	-0.742998	54505
DLD	0.2479338	7.44424882	3.60357827	0.00095321	0.08319944	-0.7626901	1738
FASTKD5	0.38234689	4.67334518	3.60303277	0.00095467	0.08319944	-0.6978787	60493
FANCA	-0.4746872	5.09372309	-3.599644	0.00096381	0.08319944	-0.7059124	2175
RSU1	0.23638654	6.42006984	3.59868364	0.00096641	0.08319944	-0.7448826	6251
CKB	-0.4871384	7.12997839	-3.5913856	0.00098643	0.08437447	-0.7762523	1152
METTL8	0.31202039	5.27495858	3.58286198	0.00101031	0.08586308	-0.7488225	79828
MTERF1	0.42141537	4.63814124	3.57463016	0.0010339	0.08624771	-0.7667273	7978
TMEM242	0.27499758	5.57948242	3.57455547	0.00103411	0.08624771	-0.776495	729515
SOAT1	0.3143222	6.11672736	3.57372386	0.00103653	0.08624771	-0.7968871	6646
NPL	0.47086764	3.2755225	3.57143242	0.0010432	0.08624771	-0.8463395	80896
GDAP2	0.26520561	5.54737936	3.57001377	0.00104736	0.08624771	-0.7870707	54834
TMEM184C	0.26307935	5.93602452	3.56627056	0.0010584	0.08651134	-0.8082917	55751
SYNM	0.43980051	6.67977256	3.56451606	0.00106361	0.08651134	-0.8393766	23336

MARS2	0.37655822	3.08104297	3.56165617	0.00107216	0.08661985	-0.8845602	92935
NARS	0.2399904	8.05189595	3.55638938	0.00108808	0.08661985	-0.8950344	4677
ABHD5	0.30917141	5.62430667	3.55595838	0.00108939	0.08661985	-0.8240181	51099
CDS2	0.17404876	7.5558165	3.55540567	0.00109108	0.08661985	-0.8890002	8760
TTC39A	0.73501064	1.82268891	3.5523766	0.00110036	0.08666267	-1.0679793	22996
PPT1	0.22271359	7.41464832	3.55097233	0.00110469	0.08666267	-0.8951358	5538
NCAPD2	-0.3246695	7.02419818	-3.5472622	0.00111621	0.08689797	-0.8970655	9918
PAQR8	0.38737066	5.50628729	3.54579374	0.0011208	0.08689797	-0.8455391	85315
TRIM16L	0.44358475	4.60885683	3.54111286	0.00113555	0.08717457	-0.8469142	147166
SAR1B	0.31353852	6.44098238	3.53930334	0.00114131	0.08717457	-0.8949292	51128
NKD1	-0.5245537	2.95355281	-3.5366562	0.00114977	0.08717457	-0.9476816	85407
LAMTOR3	0.22667142	7.04596603	3.53637826	0.00115067	0.08717457	-0.92331	8649
OSGIN2	0.29966318	5.39376556	3.53077596	0.0011688	0.0880453	-0.8793521	734
FZD2	-0.2574809	4.16765265	-3.523766	0.00119188	0.08906591	-0.8953389	2535
RPL7A	-0.2754327	6.92256485	-3.5210634	0.00120089	0.08906591	-0.9459317	6130
TAX1BP1	0.25191617	7.96685162	3.52058298	0.0012025	0.08906591	-0.9851104	8887
AEBP1	-0.6585169	5.32169758	-3.5175905	0.00121257	0.08931285	-0.9115505	165
C22orf46	0.29852609	4.86409452	3.51463597	0.0012226	0.08955356	-0.9098198	79640
STAT5B	-0.2512066	6.5041032	-3.511142	0.00123455	0.08993242	-0.9702737	6777
TUBB2B	-0.2724736	7.6234057	-3.5045495	0.00125742	0.09090008	-1.0032807	347733
E2F1	-0.443013	4.07774091	-3.5033707	0.00126155	0.09090008	-0.9441022	1869
PSMD11	0.18834915	7.48896027	3.50028869	0.00127241	0.09103879	-1.0270847	5717
H1FX	-0.6901188	4.06762698	-3.4972834	0.00128309	0.09103879	-0.9555778	8971
RPLP2	-0.4042981	7.11951122	-3.4970079	0.00128407	0.09103879	-1.0085885	6181
ASCC3	0.3021221	7.43958993	3.49333157	0.00129726	0.09148476	-1.0428737	10973
PXDN	-0.4578262	7.22353978	-3.4873621	0.00131896	0.09252267	-1.0535802	7837
PSMD12	0.31032388	6.25639293	3.48330727	0.0013339	0.09252717	-1.0257422	5718
PNO1	0.4286301	4.73091349	3.48190749	0.00133909	0.09252717	-0.9877655	56902
SREBF1	-0.5495927	4.84987841	-3.4816732	0.00133996	0.09252717	-0.9899296	6720
CBWD5	0.40023602	2.57675853	3.47740863	0.00135592	0.09314365	-1.104345	220869
MSMO1	-0.3046943	6.99889398	-3.472614	0.00137407	0.09386654	-1.0811596	6307
PIP4K2C	0.17581038	5.92885893	3.46992577	0.00138435	0.09386654	-1.0482524	79837
TMEM144	0.63953521	2.37197472	3.46905956	0.00138768	0.09386654	-1.1676453	55314

DBF4B	-0.3201537	4.08156574	-3.4651611	0.00140275	0.09428192	-1.0348536	80174
RPS8	-0.2420384	8.10941649	-3.4605831	0.00142066	0.09428192	-1.1193851	6202
SUN1	-0.2048244	7.48826281	-3.4604628	0.00142113	0.09428192	-1.1278951	23353
FDPS	-0.2283881	7.33416868	-3.4595489	0.00142473	0.09428192	-1.1218701	2224
LSG1	0.1945175	6.14616903	3.45423911	0.00144583	0.09428192	-1.0957792	55341
SAPCD2	-0.4872928	2.54279957	-3.4538248	0.00144749	0.09428192	-1.1651773	89958
ZDHHC3	0.15176343	6.4880352	3.45278745	0.00145165	0.09428192	-1.1143928	51304
FAM171A1	-0.2155839	6.25940607	-3.4516078	0.0014564	0.09428192	-1.1083161	221061
TLCD1	0.51756014	2.32907953	3.45124633	0.00145785	0.09428192	-1.2048938	116238
ZNF516	-0.2092988	5.35352063	-3.4494956	0.00146493	0.09428192	-1.0781664	9658
ATG7	0.19087325	5.5197319	3.4463283	0.00147782	0.09445595	-1.0898724	10533
ETF1	0.24926995	7.20079291	3.4433744	0.00148994	0.09445595	-1.1614712	2107
PTER	0.53281501	3.16292507	3.4427518	0.00149251	0.09445595	-1.1431124	9317
MIR137HG	0.56742002	1.6616186	3.44187367	0.00149613	0.09445595	-1.3126472	400765
PAK1IP1	0.29546887	5.76484743	3.43559842	0.0015223	0.09504004	-1.1241053	55003
SDAD1	0.27816115	6.5549142	3.43489394	0.00152527	0.09504004	-1.1595562	55153
FAM114A2	0.27994871	5.71977648	3.43082089	0.00154252	0.09504004	-1.1344089	10827
ATG16L2	-0.4925325	3.19779323	-3.4307368	0.00154287	0.09504004	-1.1548236	89849
STARD4	-0.3438458	5.47181175	-3.4300524	0.00154579	0.09504004	-1.1301736	134429
EPHX4	0.72566481	1.59455394	3.42884893	0.00155094	0.09504004	-1.3503495	253152
ASAP3	-0.3311279	6.17866003	-3.4262004	0.00156231	0.09504004	-1.1675617	55616
CDH24	-0.4920352	4.99539086	-3.425116	0.00156699	0.09504004	-1.1282858	64403
KDM2B	-0.2371719	6.42432872	-3.4244437	0.0015699	0.09504004	-1.1818982	84678
PHF21B	-0.4166702	4.95260006	-3.4216516	0.00158204	0.09533944	-1.1353624	112885
BCAP29	0.32282239	6.58067709	3.41569963	0.00160821	0.09608878	-1.2088412	55973
TEAD2	-0.2473201	5.79139379	-3.415529	0.00160897	0.09608878	-1.1765572	8463
RBM33	-0.3203277	7.16924618	-3.4132283	0.0016192	0.09626624	-1.2385044	155435
CPPED1	0.30594832	3.82112222	3.40960384	0.00163545	0.09679808	-1.1745356	55313
CCDC61	-0.5473524	1.75812453	-3.4038295	0.00166166	0.0979121	-1.358493	729440
TRERF1	-0.2520434	5.18000468	-3.3991701	0.00168309	0.09873661	-1.1946687	55809

Table S3: Effects of alcohol in the second sample (n=10) for genes with adjusted P < 0.1 in the discovery sample (n=34).

gene	logFC	AveExpr	t	P.Value	adj.P.Val	B
ABHD4	0.05458239	5.12021729	0.3493715	0.73307475	0.99723488	-4.9582383
ABHD5	0.12300348	3.91738738	1.37707789	0.19449826	0.99723488	-4.4831335
ACTG2	-0.9008248	2.66657536	-3.730734	0.00303961	0.99723488	-3.6542049
AEBP1	-0.1779748	4.42338671	-1.2115384	0.24980762	0.99723488	-4.5753881
AEN	0.25468908	4.79065033	1.92145219	0.07959733	0.99723488	-4.1148017
ALG1	0.0735911	2.80696973	0.67726491	0.5115279	0.99723488	-4.7107477
ANXA7	-0.016848	5.14563039	-0.2189191	0.83051995	0.99723488	-4.9821644
AP3M2	0.14342746	5.57081066	2.47053949	0.03009129	0.99723488	-3.6267387
AP4S1	0.06961732	4.26347715	0.64580939	0.53097908	0.99723488	-4.8119857
ASAP3	-0.0720367	5.34499919	-0.8087603	0.43493806	0.99723488	-4.8184032
ASCC3	0.08396526	7.31926915	1.31177187	0.21498179	0.99723488	-4.556772
ASF1B	-0.0925244	2.53042001	-0.603035	0.55809932	0.99723488	-4.7130801
ATG16L2	-0.2483558	3.61831723	-2.0899088	0.0593831	0.99723488	-4.1147503
ATG7	0.00065116	5.02492727	0.00852386	0.99334396	0.99874676	-4.986942
ATP6V1C1	0.1177701	6.03864076	1.94592685	0.07630968	0.99723488	-4.0463108
AURKB	-0.0509503	3.32072926	-0.5139233	0.61696043	0.99723488	-4.7759533
BCAP29	0.15630811	5.46958004	1.43575084	0.17750383	0.99723488	-4.4507801
BCAR3	0.04652444	3.68982703	0.49932667	0.62689049	0.99723488	-4.8064621
BCOR	-0.042106	6.47090752	-0.5381783	0.6006347	0.99723488	-4.9790733
BRD1	-0.1281116	5.237295	-1.6722594	0.12122156	0.99723488	-4.2805819
C21orf58	-0.2646883	3.45155865	-2.8017225	0.0164645	0.99723488	-3.7701901
C22orf46	0.02307809	4.03109809	0.19063753	0.85210736	0.99723488	-4.885204
CA12	0.20524677	5.87549492	1.49646287	0.16125497	0.99723488	-4.4091023
CBWD5	-0.0532989	3.23638791	-0.3585345	0.72638778	0.99723488	-4.7960847
CCDC61	-0.3581818	1.83376988	-2.440378	0.03177774	0.99723488	-4.2010826
CCNF	-0.0754807	3.51501854	-0.9265428	0.37305223	0.99723488	-4.6722211
CDC20	-0.1646602	3.9152514	-1.8720215	0.0866377	0.99723488	-4.2084163
CDC25B	-0.2065305	5.01623521	-2.3251574	0.0391025	0.99723488	-3.7938514

CDCA5	-0.0032949	3.77422168	-0.0230951	0.98196722	0.99852483	-4.8693367
CDCA8	-0.1369588	3.42735164	-1.0545702	0.31310637	0.99723488	-4.622254
CDH24	-0.0210523	4.46743339	-0.1782631	0.86159365	0.99723488	-4.9286376
CDS2	0.04151824	7.56479094	0.6433435	0.53252175	0.99723488	-4.9522883
CECR2	-0.2447988	6.2844063	-2.6554076	0.02150711	0.99723488	-3.4133192
CHAC2	-0.1861507	2.29823692	-1.1859208	0.25939283	0.99723488	-4.5657354
CIT	-0.1626811	5.47350152	-1.594364	0.13773834	0.99723488	-4.3358693
CKB	-0.2245364	7.3226766	-1.1215277	0.28475828	0.99723488	-4.6905206
CPPED1	0.06764376	2.90475647	0.46367343	0.65146698	0.99723488	-4.7569376
CREB3L4	-0.262566	2.49904746	-2.1650925	0.05201047	0.99723488	-4.2150001
CROT	0.02716817	4.60621029	0.20198789	0.84342741	0.99723488	-4.938803
DAZAP1	-0.1494146	5.22070166	-1.9276041	0.0787589	0.99723488	-4.0876713
DBF4B	-0.2398769	3.71509774	-2.4460231	0.03145528	0.99723488	-3.9031081
DERL3	-0.0596736	1.91309254	-0.4204564	0.68184094	0.99723488	-4.7119635
DHCR24	0.02003127	6.09733527	0.1680669	0.86942714	0.99723488	-5.0484765
DHX29	0.07348866	5.60977928	0.83454816	0.42083814	0.99723488	-4.8186428
DLD	0.05827771	5.83775196	0.6754612	0.51263177	0.99723488	-4.9006112
DLL1	0.01487995	4.00213178	0.08696556	0.932183	0.99801066	-4.8895545
DNMT3B	-0.1172832	3.53538898	-0.7894806	0.44567937	0.99723488	-4.7189403
DOLK	0.2139061	3.26945682	1.79859766	0.09814876	0.99723488	-4.299156
DTNA	-0.1011626	7.25184039	-1.4631341	0.17001084	0.99723488	-4.4409901
DUSP14	0.19323587	3.3969438	1.4610845	0.17056224	0.99723488	-4.4506454
DZIP1L	-0.1861214	3.80009429	-1.8442282	0.09084206	0.99723488	-4.2333466
E2F1	-0.1512222	3.52397451	-1.6942697	0.11688345	0.99723488	-4.3322408
EDA2R	0.19145539	4.96640966	1.72947091	0.11023211	0.99723488	-4.2450913
EFS	-0.1163884	3.68447313	-0.7381027	0.47512972	0.99723488	-4.7435252
EI24	0.16374352	5.8330913	2.28856766	0.04175083	0.99723488	-3.7619216
EMP2	-0.1632434	4.00522003	-1.1217717	0.28465867	0.99723488	-4.6096199
ENDOD1	0.1306237	5.42991347	1.2056359	0.25199093	0.99723488	-4.603831
ESPL1	-0.2397201	4.0277008	-2.5719598	0.02503536	0.99723488	-3.7709886
ETF1	0.11625536	6.16068968	1.61968681	0.13216448	0.99723488	-4.3141417

F2RL2	0.11112525	2.84120752	0.85943387	0.40752302	0.99723488	-4.6672035
FADS1	-0.1578176	7.08519647	-1.850009	0.08995256	0.99723488	-4.1170628
FAM114A2	0.02919171	4.91452638	0.28060126	0.78396115	0.99723488	-4.954937
FAM171A1	-0.0125437	5.40762462	-0.1255129	0.90226791	0.99723488	-5.0108573
FANCA	-0.325345	4.19604567	-3.9567005	0.00203312	0.99723488	-2.9417576
FASTKD5	0.22377295	4.09617255	2.17258362	0.05132525	0.99723488	-4.0096084
FBL	0.05850907	5.66573411	0.57303589	0.57756697	0.99723488	-4.9308476
FBLN1	-0.1174374	4.82706239	-0.8321119	0.42215708	0.99723488	-4.7802055
FBXO22	0.04934219	6.25145579	0.7507836	0.46775	0.99723488	-4.8846202
FDFT1	-0.0216585	6.78955362	-0.1460632	0.88638062	0.99723488	-5.0767537
FDPS	-0.0045564	4.95252163	-0.033248	0.97404242	0.99852483	-4.9806928
FOXM1	-0.1805312	3.99566944	-1.4255696	0.18035997	0.99723488	-4.4569562
FZD2	0.1947902	3.08698662	0.86559274	0.40427208	0.99723488	-4.6741877
GATB	-0.0025726	4.37432505	-0.0201228	0.98428763	0.99852483	-4.9278496
GBA	0.10431024	3.93737721	0.9095559	0.38157914	0.99723488	-4.6976251
GCLM	-0.1539726	3.79039724	-1.2608743	0.23214054	0.99723488	-4.5404804
GDAP2	-0.0472526	6.24671429	-0.8503556	0.41234709	0.99723488	-4.8356622
GPATCH4	0.07183203	3.88993648	0.86010735	0.40716667	0.99723488	-4.7138976
GPR19	0.10306815	3.08122907	0.93109037	0.37079234	0.99723488	-4.6541677
GSE1	-0.235564	7.26888687	-2.7488317	0.01813568	0.99723488	-3.2958051
GTSE1	-0.1623831	3.46227485	-1.2819128	0.2249184	0.99723488	-4.5297754
H1FX	-0.3030215	3.38614264	-1.6194706	0.13221122	0.99723488	-4.3767714
HJURP	-0.2099435	4.13984478	-1.8774166	0.08584246	0.99723488	-4.1876394
HMOX1	-0.069885	2.34255353	-0.3946105	0.70029404	0.99723488	-4.7350853
HSPH1	0.07873799	6.39254251	0.80472825	0.43717035	0.99723488	-4.8631714
IGDCC3	-0.1431116	3.96472463	-1.5845611	0.1399506	0.99723488	-4.3704452
INSIG1	-0.0707402	5.43027222	-0.3908	0.70303201	0.99723488	-4.9705344
IQGAP3	-0.2700184	4.62868112	-2.1171932	0.05660055	0.99723488	-3.9858872
KDM2B	-0.1027366	5.43595706	-1.2858659	0.22358185	0.99723488	-4.5525724
KIF18B	-0.2539112	3.46448968	-2.0629074	0.06226281	0.99723488	-4.1477528
KIF22	-0.0187578	5.0049176	-0.2403982	0.81422024	0.99723488	-4.9684221

KIF3B	0.13014707	7.12862776	1.64916703	0.12592567	0.99723488	-4.2894175
KIFC1	-0.1363857	4.51537117	-1.2867777	0.22327448	0.99723488	-4.5354203
KTN1	0.04326304	7.58600299	0.53853272	0.60039781	0.99723488	-4.9927951
LACTB2	-0.2025804	1.82656719	-1.3091387	0.21584344	0.99723488	-4.5350428
LAMTOR3	-0.0460609	5.92850294	-0.7519286	0.46708721	0.99723488	-4.8717144
LDLR	-0.1193669	5.15494504	-0.7378998	0.47524836	0.99723488	-4.8391397
LFNG	-0.2341844	3.65961357	-2.07621	0.0608281	0.99723488	-4.1175463
LSG1	0.00073911	5.12925647	0.00837202	0.99346252	0.99874676	-4.9951258
MAML1	-0.1224217	5.9071068	-2.1107848	0.05724286	0.99723488	-3.9098101
MAML3	-0.2462128	5.25536706	-3.460678	0.00494488	0.99723488	-2.9002951
MARS2	0.1228462	2.74407658	0.81233244	0.43296666	0.99723488	-4.676038
MCF2	0.1908493	2.34807574	0.89932195	0.38678127	0.99723488	-4.6426799
MDM2	0.02702661	7.42672323	0.26077697	0.79883924	0.99723488	-5.0673488
METTL8	0.01902478	4.86077778	0.21348417	0.8346578	0.99723488	-4.9599931
MFNG	-0.3359254	1.67554617	-2.0470419	0.06401534	0.99723488	-4.3330024
MFSD1	0.10031096	4.62336218	0.92784079	0.37240622	0.99723488	-4.725529
MICAL1	-0.2012728	5.53382309	-1.6750124	0.12067127	0.99723488	-4.2744708
MIR137HG	0.43268838	4.11327882	1.60363975	0.13567323	0.99723488	-4.3564385
MMAB	0.00965665	4.18738465	0.10043092	0.92171823	0.99723488	-4.9069279
MOCS2	0.08108754	5.03017734	0.82301547	0.42710592	0.99723488	-4.7953291
MSMO1	-0.0645783	6.25898133	-0.3605688	0.72490638	0.99723488	-5.0229057
MTERF1	-0.0876129	4.46188164	-0.8571052	0.40875682	0.99723488	-4.7481052
MYBL2	-0.1547539	3.93680491	-1.0785617	0.30271476	0.99723488	-4.6271559
NARS	0.10191409	6.92704508	1.75979915	0.10477516	0.99723488	-4.1957716
NCAPD2	-0.201873	5.91853308	-2.1670801	0.05182783	0.99723488	-3.8611781
NDUFAF6	-0.0212728	4.41901363	-0.2360032	0.8175483	0.99723488	-4.917484
NFS1	-0.0856846	3.50653059	-0.7863399	0.44744524	0.99723488	-4.7183022
NKD1	-0.0810446	3.83602005	-0.4411161	0.66724348	0.99723488	-4.8307158
NOV	0.08650206	2.36858168	0.30484415	0.76588848	0.99723488	-4.7454454
NPL	-0.1294261	2.20150906	-0.9611715	0.35608667	0.99723488	-4.6250658
NQO1	0.2511864	4.4556918	0.99806053	0.33862814	0.99723488	-4.6835079

NRM	-0.0706215	3.51182313	-0.7701656	0.45661062	0.99723488	-4.7235664
NXN	0.00272008	5.26330153	0.03063364	0.9760828	0.99852483	-5.0050058
OGFOD1	-0.0391456	5.11347794	-0.6729662	0.51416105	0.99723488	-4.8625042
OSGIN2	0.04517589	4.7301817	0.39013062	0.70351344	0.99723488	-4.9188582
OSTM1	0.18479324	4.74810383	1.98110077	0.07180396	0.99723488	-4.0742846
PAK1IP1	0.08653238	3.96210533	0.76273708	0.46085991	0.99723488	-4.7525128
PAPD7	-0.1652523	5.29994187	-2.2786734	0.04249597	0.99723488	-3.8052455
PAQR8	0.13361286	4.61705326	1.02638171	0.32565307	0.99723488	-4.6777353
PARN	0.05971918	5.02950632	0.80644751	0.43621759	0.99723488	-4.8026762
PEX3	-0.0123073	4.10853898	-0.1303467	0.89852671	0.99723488	-4.8974546
PFDN1	0.20152406	4.93574803	2.50051648	0.02850146	0.99723488	-3.669961
PHF21B	-0.161616	4.60292671	-2.1350293	0.05484842	0.99723488	-3.975015
PIF1	-0.2193335	2.24550571	-1.6001787	0.1364406	0.99723488	-4.4369982
PINX1	0.27605859	1.78552325	1.53012049	0.15280709	0.99723488	-4.4755797
PIP4K2C	-0.0576353	4.56158034	-0.7780877	0.45210655	0.99723488	-4.7865421
PLEKHH2	-0.060353	3.55585795	-0.245994	0.80998836	0.99723488	-4.8362662
PLK1	0.02979985	3.5102876	0.29330451	0.77447383	0.99723488	-4.8269117
PNO1	0.03895348	2.96853719	0.2691251	0.7925635	0.99723488	-4.7864577
POC1A	-0.2621663	2.54058274	-2.2772713	0.04260259	0.99723488	-4.1672193
PPT1	0.04308353	6.29211656	0.48867976	0.6341824	0.99723488	-4.9888625
PRR11	-0.1058519	3.58977131	-0.7946293	0.4427942	0.99723488	-4.7203054
PSMD11	-0.0836573	5.86291644	-1.2644937	0.23088491	0.99723488	-4.575126
PSMD12	0.03526405	5.9553095	0.45226792	0.65942249	0.99723488	-4.9845681
PTCHD4	0.21543863	4.8474916	1.95447208	0.07519163	0.99723488	-4.0872614
PTMA	0.05636112	8.36193327	0.71708227	0.48752108	0.99723488	-4.9118237
PTMS	-0.0853245	6.98456475	-0.9432147	0.36481438	0.99723488	-4.7998891
PXDN	-0.0977751	6.75926353	-0.6490851	0.5289338	0.99723488	-4.9441242
RAD54L	-0.3655202	2.96159453	-2.4794234	0.02961136	0.99723488	-4.022477
RBM33	-0.0987218	7.2527438	-1.5730804	0.1425808	0.99723488	-4.3525137
RCC2	-0.0683308	5.65614878	-0.8200002	0.42875479	0.99723488	-4.8279888
RCOR1	-0.1658606	5.40601111	-1.8411686	0.09131608	0.99723488	-4.1483208

RCOR2	-0.1920185	4.96965032	-1.1080216	0.29031293	0.99723488	-4.6486421
RNF122	-0.1046128	3.27471089	-0.7973878	0.44125344	0.99723488	-4.7029183
RNF185	0.11386311	4.93726017	1.37485578	0.19516767	0.99723488	-4.4872363
RPL10	0.09050719	8.3739036	1.05979677	0.31082012	0.99723488	-4.7248109
RPL7A	0.09855002	9.05106123	1.07821006	0.30286519	0.99723488	-4.705068
RPLP2	0.08402706	7.12803819	0.71518113	0.48865147	0.99723488	-4.9198131
RPS11	-0.0187594	8.87960328	-0.1948355	0.84889463	0.99723488	-5.0562417
RPS8	0.13698304	8.71180445	1.63402856	0.12909623	0.99723488	-4.3022304
RRM2B	0.10804967	5.61000142	1.1730133	0.2643306	0.99723488	-4.6290168
RSU1	0.12142075	4.86751466	1.3937073	0.18954904	0.99723488	-4.4745903
SAR1B	0.00330049	5.6689862	0.04801913	0.96251836	0.99852483	-5.0324945
SCD	-0.1905985	8.88827002	-0.9570118	0.35809505	0.99723488	-4.7799327
SCFD2	0.06290854	4.37018142	0.75246208	0.46677861	0.99723488	-4.7831974
SDAD1	0.11491419	4.61142289	1.17137228	0.26496361	0.99723488	-4.6018359
SERPINB8	-0.0411108	2.59425733	-0.2690855	0.79259322	0.99723488	-4.7621197
SHMT1	-0.148651	3.59713313	-1.4467357	0.17446514	0.99723488	-4.4524603
SLC25A20	0.23841324	2.80254395	1.30656999	0.21668669	0.99723488	-4.5240598
SMAD9	-0.1929766	5.46428261	-2.2702785	0.04313816	0.99723488	-3.7999094
SMOC1	-0.1407756	5.44862218	-1.0427621	0.31831768	0.99723488	-4.7023791
SNAPC5	-0.0255536	3.22774824	-0.2523316	0.80520309	0.99723488	-4.8078906
SNRPA	0.00292733	4.13498361	0.0288849	0.9774477	0.99852483	-4.9040623
SOAT1	0.01242991	5.11500569	0.11467525	0.91066481	0.99723488	-4.9901166
SPRED3	-0.0408997	3.92145292	-0.2904819	0.77657867	0.99723488	-4.8636038
SREBF1	-0.3004279	4.78656286	-1.9947621	0.07012141	0.99723488	-4.0613555
STAMBP	-0.0300306	5.45097985	-0.4457186	0.66401062	0.99723488	-4.9578858
STARD4	-0.1126637	4.90944026	-0.6774741	0.51139998	0.99723488	-4.8479596
STAT5B	-0.1213927	5.72724671	-1.6301724	0.12991499	0.99723488	-4.3073215
SUN1	-0.0899859	6.65645396	-1.4321247	0.17851666	0.99723488	-4.4632578
SYNM	0.08679633	5.30762887	0.51420846	0.61676719	0.99723488	-4.9287285
TACC3	-0.3433905	4.01061815	-3.5282829	0.00437542	0.99723488	-3.2343391
TAX1BP1	-0.0276008	6.6904408	-0.361642	0.7241253	0.99723488	-5.0376901

TEAD2	0.07495658	4.54665008	0.85556594	0.40957371	0.99723488	-4.7534718
TGIF2	-0.0717367	4.27348823	-0.7325593	0.47837835	0.99723488	-4.784094
TIGAR	-0.1223543	3.48834308	-0.8213112	0.42803734	0.99723488	-4.7063824
TK1	-0.3136949	2.00390269	-2.2271008	0.04659021	0.99723488	-4.247678
TLCD1	0.1479277	1.79345928	1.00755975	0.33423478	0.99723488	-4.6088347
TM7SF3	0.05258052	5.55834799	0.92225172	0.37519351	0.99723488	-4.7722908
TMC7	-0.1906317	2.04937177	-1.6833607	0.11901601	0.99723488	-4.4190099
TMEM184C	0.06455791	5.85834473	1.14611955	0.27485425	0.99723488	-4.6526151
TMEM199	0.11123205	2.67663809	1.10060034	0.29340005	0.99723488	-4.5929745
TMEM242	0.08291852	4.28728189	1.1422257	0.27640447	0.99723488	-4.6077083
TOMM34	-0.0060697	4.06708635	-0.0698973	0.9454667	0.99801066	-4.8964258
TOR1A	0.05706864	4.6352731	0.83016606	0.42321248	0.99723488	-4.7695438
TRERF1	-0.2226236	5.09356097	-2.1522602	0.05320443	0.99723488	-3.9209384
TRIM16L	0.17880588	3.23903436	1.32696259	0.21006572	0.99723488	-4.5118278
TROAP	-0.2042161	3.03025264	-1.9256904	0.07901884	0.99723488	-4.2594139
TTC39A	0.29546814	1.81506562	1.93547126	0.07769854	0.99723488	-4.3555476
TUBB2B	0.0707749	8.74323072	0.59781221	0.5614625	0.99723488	-4.9551577
TXNRD1	0.09938385	7.0997474	1.27475116	0.22735619	0.99723488	-4.5833479
UBXN8	0.22394684	2.74263032	1.61580301	0.13300631	0.99723488	-4.4098466
UCP2	-0.3201872	3.28415195	-1.550022	0.14799344	0.99723488	-4.413341
UPP1	-0.1314189	3.76783256	-1.0271272	0.32531653	0.99723488	-4.6434988
UTP15	0.10621237	4.38285901	0.9477415	0.36259999	0.99723488	-4.7038919
VPS33A	-0.0193274	3.35997577	-0.150114	0.88325485	0.99723488	-4.8271774
ZCCHC17	0.06268174	5.04194689	0.84622516	0.4145546	0.99723488	-4.7855066
ZDHHC3	0.12803866	5.45144592	1.90791641	0.08147109	0.99723488	-4.094559
ZMAT3	-0.0076377	7.02087124	-0.0720167	0.94381622	0.99801066	-5.0862254
ZNF395	-0.1429864	5.21659495	-1.5097857	0.1578643	0.99723488	-4.3978401
ZNF436	0.20459742	6.60220567	2.4872885	0.02919267	0.99723488	-3.549472
ZNF516	-0.1746897	5.56577455	-2.0385333	0.06497409	0.99723488	-3.984403

Table S4: Ingenuity Pathway Analysis results for 226 genes differential expressed (adj p<0.1) after alcohol treatment

Analysis	Ingenuity Canonical Pathways	-log (B-H p-value)	Molecules
Alcohol vs Sham	Notch Signaling	1.16	*DLL1, MFNG, MAML1, MAML3, LFNG*
	Superpathway of Cholesterol Biosynthesis	1.16	*DHCR24, FDPS, FDFT1, MSMO1*
	Cholesterol Biosynthesis I	1.16	*DHCR24, FDFT1, MSMO1*
	Cholesterol Biosynthesis II (via 24,25-dihydrolanosterol)	1.16	*DHCR24, FDFT1, MSMO1*
	Cholesterol Biosynthesis III (via Desmosterol)	1.16	*DHCR24, FDFT1, MSMO1*
	Molybdenum Cofactor Biosynthesis	1.13	MOCS2, NFS1

Table S5: Summary of modules identified by WGCNA (module/gene count):

brown	cyan	darkgreen	darkgrey	darkmagenta	darkorange	darkturquoise
3263	232	217	1716	60	400	151
lightgreen	midnightblue	orangered4	paleturquoise	red	royalblue	skyblue
283	359	42	3154	1828	178	126
grey60	greenyellow	yellowgreen**	skyblue3			
811	328	**58**	52			

** decreases in alcohol treated cells p<0.001

Table S6: Genes in yellowgreen module

gene	membership	gene	membership
ARHGEF39	0.743953076	KIF22	0.92453865
ASF1B	0.89193853	KIFC1	0.928161956
AURKB	0.974749021	MCM2	0.904969869
C21orf58	0.838376168	MCM3	0.852510082
CDC20	0.954961682	MCM7	0.948318297
CDC45	0.91401219	MYBL2	0.945475277
CDCA3	0.833462536	MYCN	0.769571778
CDCA4	0.814043294	NCAPD2	0.749928564
CDCA5	0.853455824	NDE1	0.806605781
CDCA8	0.857739197	NONO	0.806882912
CDT1	0.837298273	NRM	0.773367358
CHAF1A	0.81998896	PARP1	0.786273338
CHAF1B	0.826230711	PIF1	0.929145697
DHCR24	0.820146725	PLK1	0.84276731
E2F1	0.901113741	POC1A	0.87342334
E2F2	0.752293618	POLA2	0.746839815
ESPL1	0.859306492	RAD54L	0.924738568
FADS1	0.744849359	RNASEH2A	0.840665594
FANCA	0.789227467	SAPCD2	0.88874682
FOXM1	0.845252674	SLC6A16	0.701173917
FOXN4	0.757591993	SNRPA	0.785469483
GINS4	0.661099946	SSRP1	0.751777582
GTSE1	0.941642035	TACC3	0.916765042
H2AFX	0.620266751	TAF15	0.643591361
H2AFY2	0.765803384	TIMELESS	0.798186805
HAUS8	0.763078227	TRAIP	0.873052717
HJURP	0.871932845	TROAP	0.910704987
IQGAP3	0.755654147	TUBB	0.765478811
KIF18B	0.941021231	UHRF1	0.853719364

Table S7: Previously identified eQTLs from cortex tissue have a greater effect than expected by chance in neural cell cultures derived from iPSC

Tissue	GTEx eQTL SNPs (total N)	GTEx SNPs examined in neural cell cultures (N)	set test pvalue
Anterior cingulate cortex	22240	38	0.001053999
Cortex	48499	94	0.002554997
Frontal cortex	39336	82	0.011917988
Hypothalamus	22706	40	0.084847915
Hippocampus	20792	30	0.08979091
Nucleus accumbens	39369	69	0.105491895
All tissue	1622952	1415	0.168880831
Caudate	47653	90	0.169426831
Cerebellum	83526	139	0.193147807
Cerebellar Hemisphere	62462	99	0.278973721
Putamen	28919	71	0.335778664
Whole blood	140265	185	0.8002852

Table S8: Results for the alcohol dehydrogenase gene family.

Experiment	In primary analysis	gene	logFC	**AveExpr**	t	P.Value*	adj.P.Val	B	entrezID
1	yes	ADH5	0.17564864	**7.7859347**	2.90312272	0.00667652	0.2046167	-2.709071	128
1	yes	ADH6	-0.0137589	**1.2715945**	-0.0689021	0.9455008	1	-5.491002	130
1	no	ADH4	0.7577773	**-0.4532981**	2.30493645	0.02788045	0.3208303	-3.415705	127
1	no	ADH1A	-0.1262146	**-1.2344769**	-0.548478	0.58721023	0.9056443	-5.187685	124
1	no	ADH1B	-0.5228393	**-1.6804911**	-0.7817662	0.44015856	0.8114221	-5.075818	125
1	no	ADH1C	0.31164238	**-2.6641545**	0.53445243	0.59676145	0.9107778	-5.188312	126
1	no	ADH7	0.91042325	**-4.6469406**	2.68914861	0.01132869	0.241027	-2.788425	131
2	yes	ADH5	0.08008847	**7.1957102**	1.4448705	0.18386133	1	-5.271859	
2	yes	ADH6	-0.2140017	**2.3502246**	-1.4979912	0.16984627	1	-4.666375	
2	yes	ADH4	-0.1415701	**1.865277**	-0.7253654	0.48746026	1	-5.296505	
2	no	ADH1A	-0.3725636	**0.6515676**	-3.4733943	0.00749308	1	-2.452529	
2	no	ADH1B	0.1514567	**-1.176607**	0.3275074	0.75110121	1	-5.077599	
2	no	ADH1C	-1.0705314	**-2.7087372**	-3.2181571	0.01113225	1	-3.144361	
2	no	ADH7	0.17716793	**-4.0954803**	0.4556209	0.65992726	1	-4.949128	

* these analyses did not have an expression threshold and included all genes. Note that the p value is slightly different compared to the main analysis.

Table S9. DAVID analysis of alcohol responsive 58 gene network ('yellowgreen' module). Categories with FDR < 5% are shown.

Category	Term	Count	%	PValue	Fold Enrichment	Bonferroni	Benjamini	FDR
KEGG_PATHWAY	hsa04110:Cell cycle	9	1.55E+01	1.52E-07	1.30E+01	5.93E-06	5.93E-06	1.38E-04
KEGG_PATHWAY	hsa03030:DNA replication	5	8.62E+00	3.80E-05	2.42E+01	1.48E-03	7.40E-04	3.45E-02
GOTERM_BP_DIRECT	GO:0006260~DNA replication	12	2.07E+01	6.29E-11	1.70E+01	2.31E-08	2.31E-08	8.66E-08
GOTERM_BP_DIRECT	GO:0051301~cell division	13	2.24E+01	2.12E-08	8.49E+00	7.77E-06	3.88E-06	2.92E-05
GOTERM_BP_DIRECT	GO:0006281~DNA repair	11	1.90E+01	4.74E-08	1.07E+01	1.74E-05	5.80E-06	6.53E-05
GOTERM_BP_DIRECT	GO:0006270~DNA replication initiation	6	1.03E+01	2.16E-07	4.28E+01	7.93E-05	1.98E-05	2.98E-04
GOTERM_BP_DIRECT	GO:0000082~G1/S transition of mitotic cell cycle	8	1.38E+01	2.50E-07	1.78E+01	9.17E-05	1.83E-05	3.44E-04
GOTERM_BP_DIRECT	GO:0007049~cell cycle	9	1.55E+01	3.14E-06	9.65E+00	1.15E-03	1.92E-04	4.33E-03
GOTERM_BP_DIRECT	GO:0007062~sister chromatid cohesion	7	1.21E+01	4.46E-06	1.58E+01	1.64E-03	2.34E-04	6.14E-03
GOTERM_BP_DIRECT	GO:0007067~mitotic nuclear division	8	1.38E+01	9.62E-05	7.23E+00	3.47E-02	4.40E-03	1.32E-01
GOTERM_BP_DIRECT	GO:0000070~mitotic sister chromatid segregation	4	6.90E+00	1.90E-04	3.45E+01	6.74E-02	7.73E-03	2.62E-01
GOTERM_BP_DIRECT	GO:0007080~mitotic metaphase plate congression	4	6.90E+00	5.93E-04	2.37E+01	1.96E-01	2.15E-02	8.13E-01
GOTERM_BP_DIRECT	GO:0006335~DNA replication-dependent nucleoson	3	5.17E+00	6.07E-04	7.76E+01	2.00E-01	2.01E-02	8.33E-01
GOTERM_BP_DIRECT	GO:0032508~DNA duplex unwinding	4	6.90E+00	9.46E-04	2.02E+01	2.93E-01	2.85E-02	1.30E+00
GOTERM_BP_DIRECT	GO:0006334~nucleosome assembly	4	6.90E+00	1.16E-03	1.88E+01	3.48E-01	3.23E-02	1.59E+00
GOTERM_BP_DIRECT	GO:0000086~G2/M transition of mitotic cell cycle	5	8.62E+00	3.49E-03	7.84E+00	7.22E-01	8.75E-02	4.70E+00
GOTERM_CC_DIRECT	GO:0005654~nucleoplasm	32	5.52E+01	7.93E-09	2.76E+00	8.16E-07	8.16E-07	8.85E-06
GOTERM_CC_DIRECT	GO:0000790~nuclear chromatin	9	1.55E+01	3.51E-07	1.29E+01	3.62E-05	1.81E-05	3.92E-04
GOTERM_CC_DIRECT	GO:0005634~nucleus	38	6.55E+01	1.81E-06	1.91E+00	1.87E-04	6.22E-05	2.02E-03
GOTERM_CC_DIRECT	GO:0000785~chromatin	6	1.03E+01	3.98E-05	1.53E+01	4.09E-03	1.02E-03	4.44E-02
GOTERM_CC_DIRECT	GO:0000784~nuclear chromosome, telomeric regior	6	1.03E+01	6.88E-05	1.36E+01	7.06E-03	1.42E-03	7.68E-02
GOTERM_CC_DIRECT	GO:0005730~nucleolus	12	2.07E+01	5.33E-04	3.41E+00	5.35E-02	9.11E-03	5.94E-01
GOTERM_CC_DIRECT	GO:0042555~MCM complex	3	5.17E+00	7.48E-04	7.05E+01	7.42E-02	1.09E-02	8.32E-01
GOTERM_CC_DIRECT	GO:0005871~kinesin complex	4	6.90E+00	1.18E-03	1.88E+01	1.14E-01	1.50E-02	1.30E+00
GOTERM_CC_DIRECT	GO:0000922~spindle pole	5	8.62E+00	1.27E-03	1.04E+01	1.23E-01	1.44E-02	1.41E+00
GOTERM_CC_DIRECT	GO:0005813~centrosome	8	1.38E+01	2.40E-03	4.23E+00	2.20E-01	2.45E-02	2.65E+00
GOTERM_CC_DIRECT	GO:0005657~replication fork	3	5.17E+00	2.76E-03	3.73E+01	2.48E-01	2.55E-02	3.04E+00
GOTERM_CC_DIRECT	GO:0051233~spindle midzone	3	5.17E+00	3.45E-03	3.34E+01	2.99E-01	2.92E-02	3.78E+00
GOTERM_CC_DIRECT	GO:0043234~protein complex	7	1.21E+01	3.50E-03	4.64E+00	3.03E-01	2.74E-02	3.84E+00
GOTERM_MF_DIRECT	GO:0042393~histone binding	7	1.21E+01	2.51E-06	1.74E+01	2.89E-04	2.89E-04	2.86E-03
GOTERM_MF_DIRECT	GO:0003678~DNA helicase activity	4	6.90E+00	1.86E-04	3.48E+01	2.12E-02	1.07E-02	2.12E-01
GOTERM_MF_DIRECT	GO:0003677~DNA binding	17	2.93E+01	2.13E-04	2.73E+00	2.42E-02	8.15E-03	2.43E-01
GOTERM_MF_DIRECT	GO:0005515~protein binding	45	7.76E+01	6.39E-04	1.36E+00	7.08E-02	1.82E-02	7.25E-01
GOTERM_MF_DIRECT	GO:0003682~chromatin binding	8	13.7931034	7.62E-04	5.14882155	0.08390196	0.01737368	0.86424249

Table S10: The effects of genes reported by McClintik et al. 2018 in neural cultures derived from iPSC.

Tissue	Direction induced by alcohol	Total Genes	Genes analyzed in iPSC	set test pvalue*
Ventral Hippocampus	Up	87	64	6.816E-02
	Down	329	251	1.000E-06
Prefrontal Cortex	Up	414	313	9.999E-01
	Down	223	175	2.202E-01
Cross-tissue effects	Up	10	8	2.200E-02
	Down	1	1	NS

*directional test of differentially expressed rat genes in alcohol treated iPSC neural cultures based on 1,000,000 simulations
