# Supplementary material for: Alcohol-responsive genes identified in human iPSC-derived neural cultures
Source: Transl Psychiatry. 2019 Mar 12;9:96. doi: 10.1038/s41398-019-0426-5 (PMC6414668; doi:10.1038/s41398-019-0426-5)
Supplement: Supplementary file 2 — SUPPLEMENTAL FIGURES [file 41398_2019_426_MOESM2_ESM.docx]

Alcohol-responsive genes identified in human iPSC-derived neural cultures

Kevin P. Jensen, Richard Lieberman, Henry R. Kranzler, Joel Gelernter, Kaitlin Clinton, and Jonathan Covault

**Supplemental Figures:**


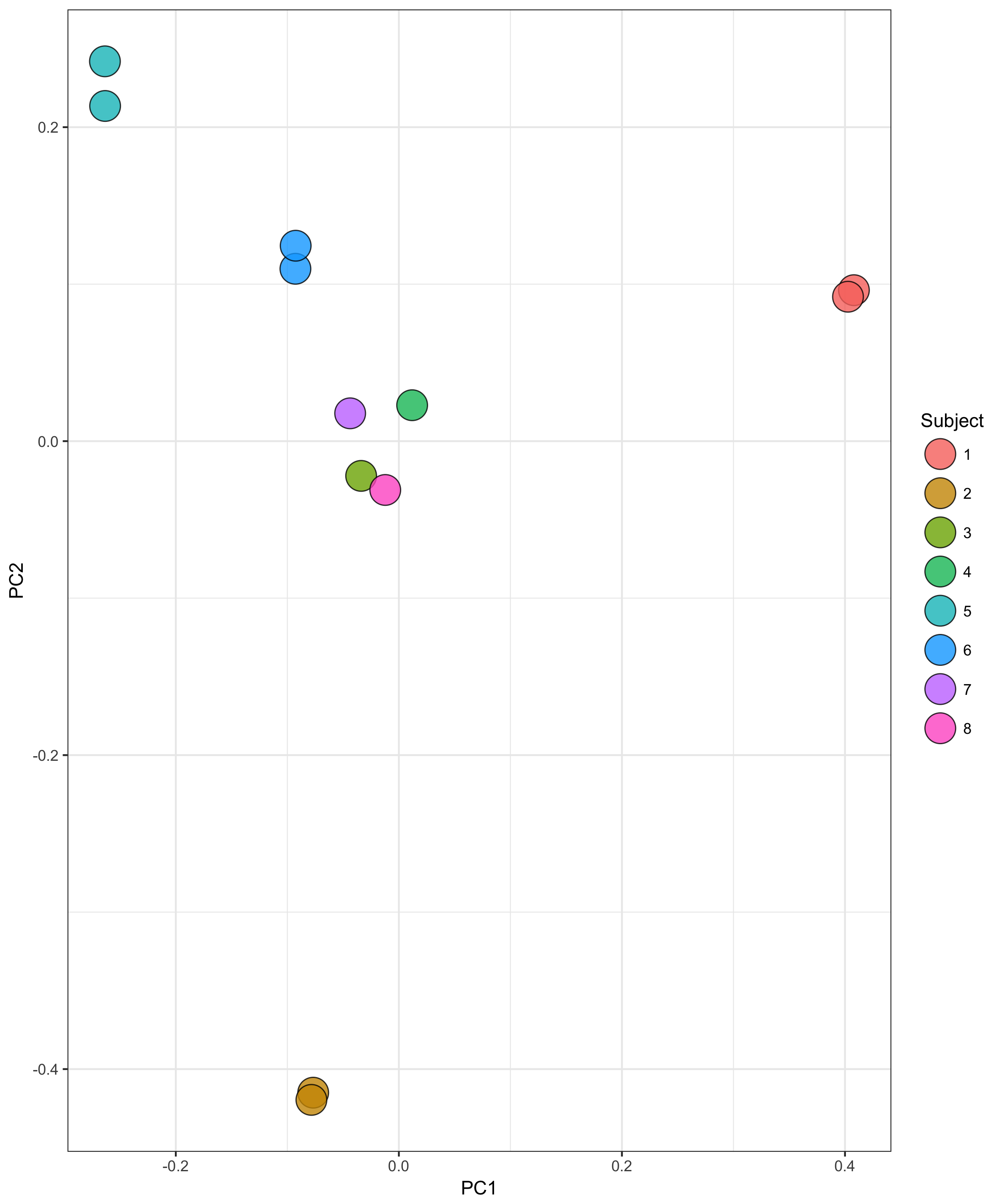


**Figure S1**: Clustering of subject genetic data.

Principal component analysis of autosomal SNPs (n = 14,770) with minor allele frequency > 20% and genotyped in > 50% of the samples. Each point represents the SNP calls from sham- and alcohol-treated sample pairs (The diagnostic group (alcohol dependent vs controls) and exposure conditions are not expected to have effects on the relative similarities of SNP calls between samples from different vs. the same subject). Four subjects (1,2,5,6) were represented by 2 samples and four other subjects (3,4,7,8) were represented by 1 sample. Note that samples from the same subjects (1,2,5,6) cluster more tightly than samples from different subjects (3,4,7,8).

**Figure S2:** eQTLs identified using neural cell culture RNA seq data.

Alleles are coded additively as 0,1 or 2 along the x-axis. The association of rs2006748 to *ZNF18* expression was significant at an FDR threshold of 5%, while all other SNP gene associations shown are significant at an FDR threshold of 10%.


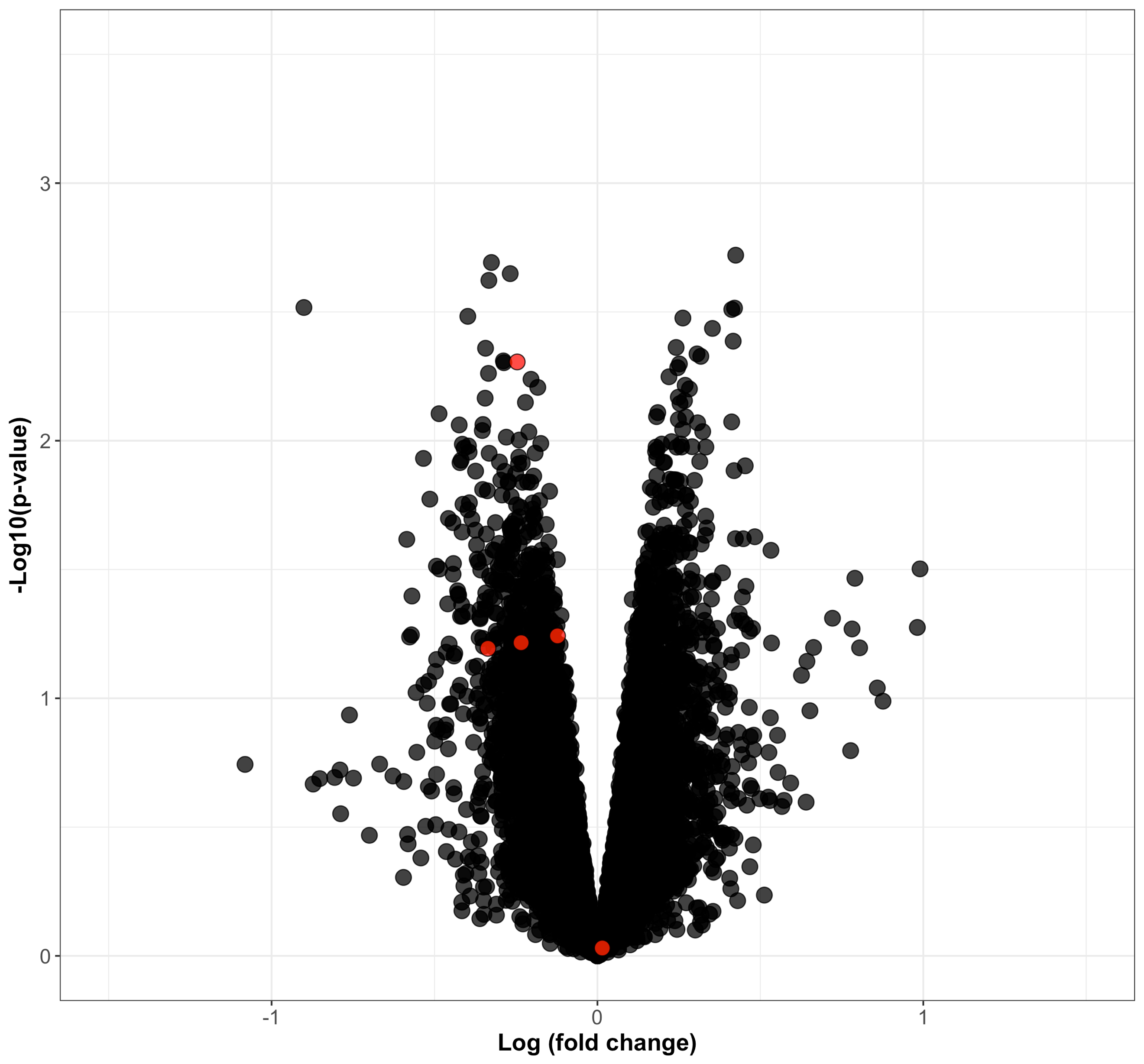


**Figure S3:** The effects of alcohol treatment on the expression of genes in the “Notch Signaling” pathway in the second experiment (n=10), which had intermittent exposure to the same concentration of alcohol as the primary sample.


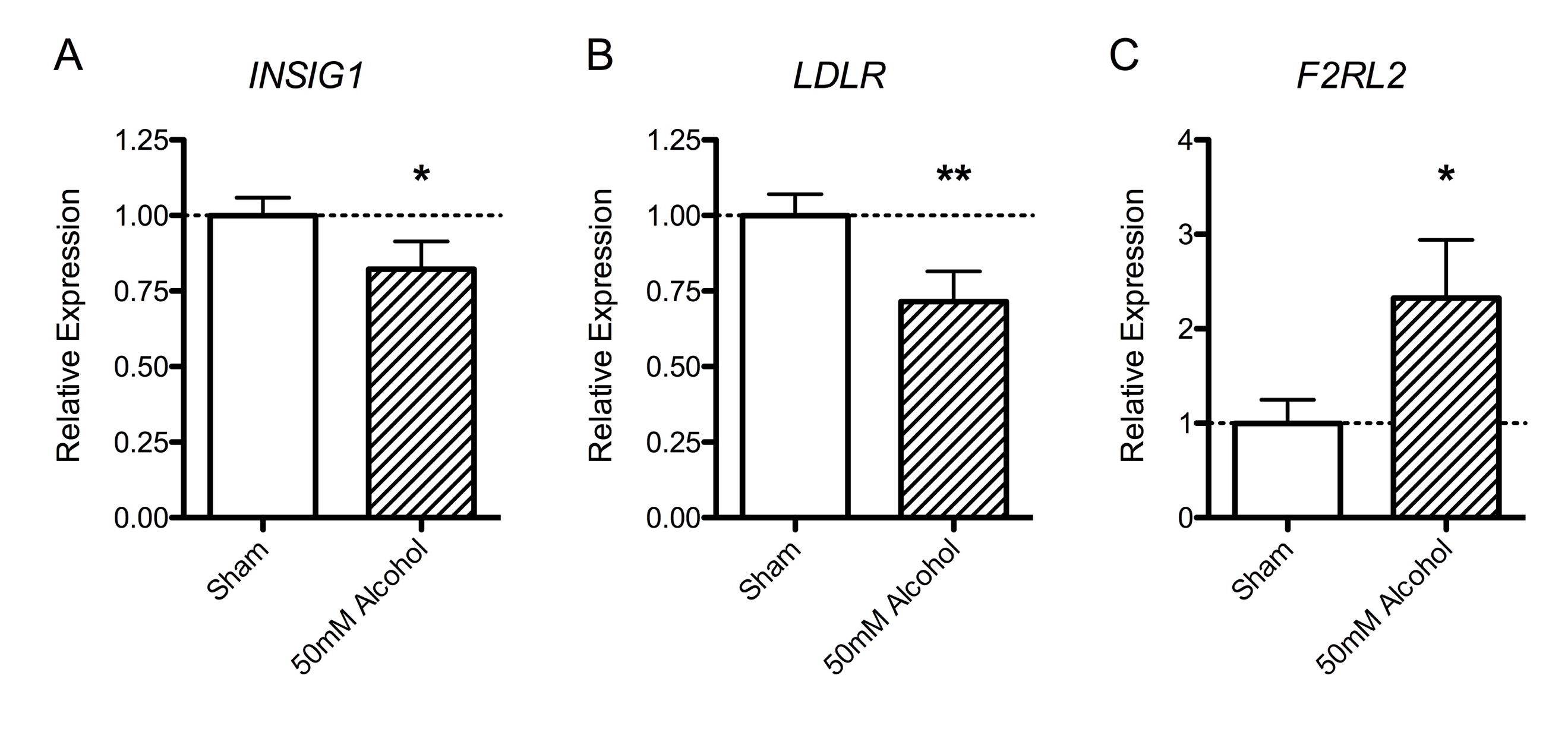


**Figure S4:** qPCR validation of the top three alcohol-responsive genes identified via RNA sequencing. 12-week-old neural cultures derived from 12 donor subjects (7 control and 5 AD) were exposed to 50mM alcohol for 7 days, the same treatment paradigm used for the batch 1 and batch 2 samples submitted for RNA sequencing. In this validation experiment, RNA from 1 control and 1 AD donor was the same input used for RNA sequencing. Paired t-tests revealed significant decreases in (A) *INSIG1* and (B) *LDLR* and increased (C) *F2RL2* mRNA expression in alcohol exposed cultures compared to sham treatment. The direction of the expression change was consistent for all three genes in the primary and validation cohorts. Statistical analysis was performed on gene expression relative to a standard curve. Data was normalized to the sham condition for visual representation. *= p<0.05, **= p<0.01


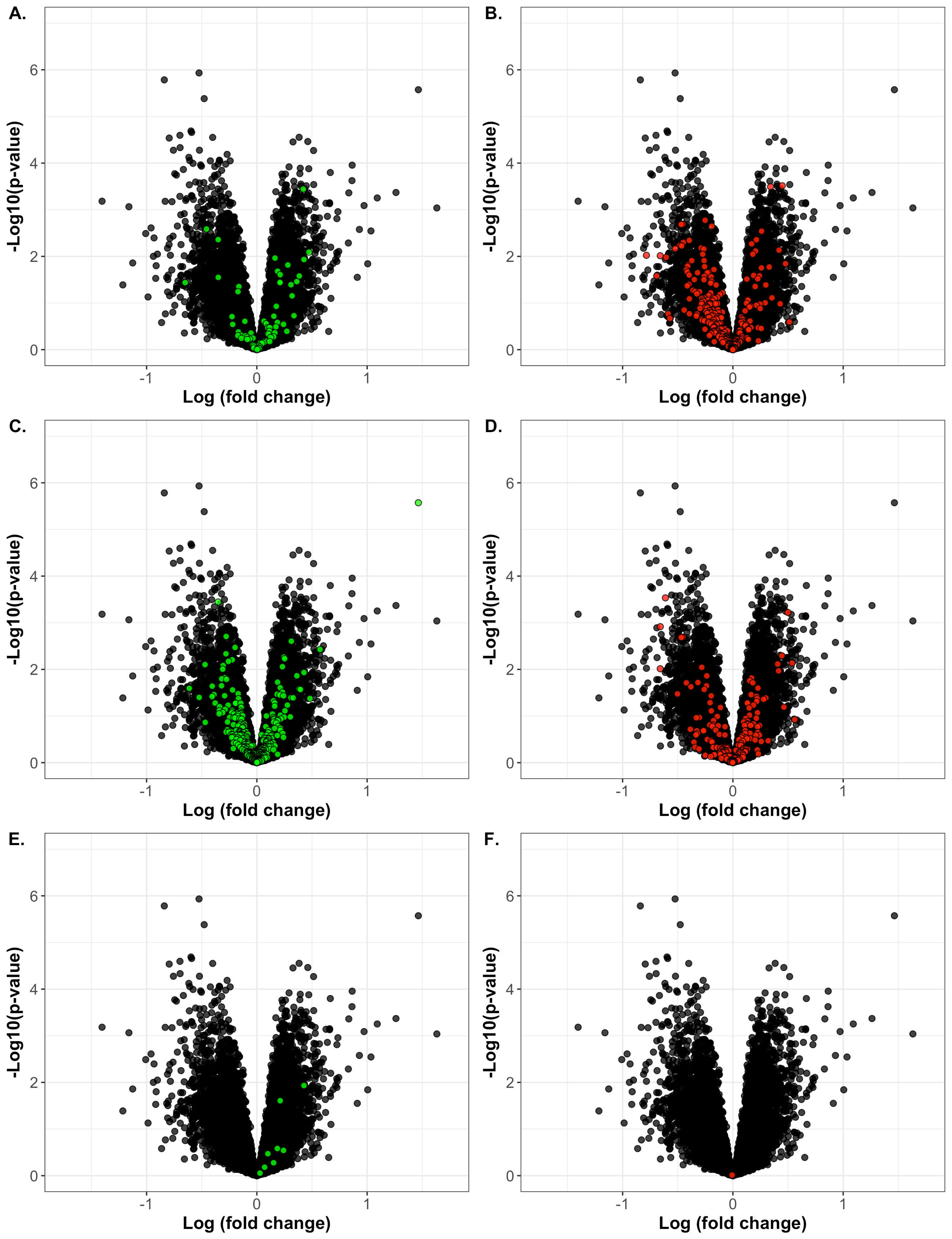


**Figure S5:** The effects of alcohol exposure on neural cell cultures derived from iPSCs for rat genes that were up (green) or down (red) regulated in response to alcohol in ventral hippocampus (**A, B**), prefrontal cortex (**C, D**) and at least 4 out of 11 rat brain regions (**E, F**) based on the study by McClintick et al.^37^.

**Figure S6.** Weighted gene co-expression network analysis steps. **A)** Estimation of soft-power for signed network construction. **B)** Clustering dendrogram of genes with dissimilarity based on topological overlap and **C)** assignment of genes in the clustering to modules. **D)** Clustering of model eigengenes and **E)** threshold for merging closely related modules**. F)** Clustering dendrogram with original and merged gene modules assignments.

**Figure S7**. Module membership is correlated with the response to alcohol.

Genes that were more tightly associated with the module (x-axis) decreased more in the alcohol condition (y-axis, correlation of gene expression with treatment) than genes that were less tightly associated to the module
